# Effectiveness and cost-effectiveness of online recorded recovery narratives in improving quality of life for people with psychosis experience (NEON Trial): a pragmatic randomised controlled trial

**DOI:** 10.1016/j.lanepe.2024.101101

**Published:** 2024-10-23

**Authors:** Mike Slade, Stefan Rennick-Egglestone, Clare Robinson, Chris Newby, Rachel A. Elliott, Yasmin Ali, Caroline Yeo, Tony Glover, Sean P. Gavan, Luke Paterson, Kristian Pollock, Stefan Priebe, Graham Thornicroft, Jeroen Keppens, Melanie Smuk, Donna Franklin, Rianna Walcott, Julian Harrison, Dan Robotham, Simon Bradstreet, Steve Gillard, Pim Cuijpers, Marianne Farkas, Dror Ben-Zeev, Julie Repper, Yasuhiro Kotera, James Roe, Joy Llewellyn-Beardsley, Fiona Ng

**Affiliations:** a School of Health Sciences, Institute of Mental Health, University of Nottingham, Nottingham, UK; b Health and Community Participation Division, Faculty of Nursing and Health Sciences, Nord University, Namsos, Norway; c Centre for Evaluation and Methods, Wolfson Institute of Population Health, Pragmatic Clinical Trials Unit, Queen Mary University of London, London, UK; d School of Medicine, University of Nottingham, Nottingham, UK; e Manchester Centre for Health Economics, Division of Population Health, Health Services Research & Primary Care, University of Manchester, Manchester, UK; f Department of Architecture and Built Environment, Faculty of Engineering, University of Nottingham, UK; g DRT Software, Nottingham, UK; h School of Health Sciences, University of Nottingham, Nottingham, UK; i Unit for Social and Community Psychiatry, East London NHS Foundation Trust, London, UK; j Centre for Implementation Science and Centre for Global Mental Health, Health Service and Population Research Department, Institute of Psychiatry, Psychology & Neuroscience, King's College London, London, UK; k Department of Informatics, King's College London, London, UK; l Centre for Genomics and Child Health, Blizard Institute, Queen Mary University of London, London, UK; m NEON Lived Experience Advisory Panel, Nottingham, UK; n School of Health and Related Research, University of Sheffield, Sheffield, UK; o Black Communication and Technology Lab, Department of Communication, University of Maryland, College Park, MD, USA; p McPin Foundation, London, UK; q Institute of Health and Wellbeing, University of Glasgow, Glasgow, UK; r School of Health Sciences, City, University of London, London, UK; s Department of Clinical, Neuro and Developmental Psychology, Amsterdam Public Health Research Institute, Vrije Universiteit Amsterdam, Amsterdam, the Netherlands; t Babeș-Bolyai University, International Institute for Psychotherapy, Cluj-Napoca, Romania; u Center for Psychiatric Rehabilitation, College of Health and Rehabilitation Sciences, Boston University, Boston, MA, USA; v Behavioral Research in Technology and Engineering (BRiTE) Center, School of Medicine, University of Washington, Seattle, WA, USA; w ImROC, Nottingham, UK; x Center for Infectious Disease Education and Research, Osaka University, Osaka, Japan; y National Institute for Health and Care Research (NIHR) Applied Research Collaboration East Midlands, University of Nottingham, Nottingham, UK

**Keywords:** Recovery narrative, Lived experience narrative, Autobiography, Digital health intervention, Digital health technology, Online trial

## Abstract

**Background:**

The Narrative Experiences Online (NEON) Intervention provides self-managed web-based access to mental health recovery narratives (n = 659). We evaluated effectiveness and cost-effectiveness in improving quality of life for adults resident in England with mental health problems and recent psychosis experience.

**Methods:**

Prospectively registered pragmatic parallel-group randomised trial controlling for usual care, recruiting from statutory mental health services and through community engagement activities, with a 52-week primary endpoint (ISRCTN11152837). All trial procedures and the NEON Intervention were delivered by an integrated web-application. Randomisation was through an independently generated list (no stratification). Allocation was masked for statistical staff and the Chief Investigator but not participants. Intervention arm participants received immediate NEON Intervention access. Control arm participants received access after completing primary endpoint questionnaires. The primary outcome was quality of life through the Manchester Short Assessment (MANSA). Serious Adverse Events (SAEs) were collected through web-based safety report forms and identified from health service usage data. The primary analysis was by a prospectively described Intention To Treat principle excluding participants who had registered multiple times, with multiple imputation for missing data.

**Findings:**

Between 9 March 2020 and 1 March 2021, 739 participants were randomised (intervention:370; control: 369), providing more than 90% power to detect a baseline-adjusted difference of 0.25 in the MANSA score. Mean age was 34.8 years (standard deviation (SD) 12.0), 561 (75.9%) were white British, 443 (59.9%) were female, 609 (82.4%) had accessed specialist care mental health services, and 698 (94.5%) had accessed primary care mental health services. Mean baseline MANSA score was 3.7 for control and intervention arms (SD 0.9 and 1.0). 565 (76.5%) participants provided primary endpoint MANSA data with a mean score of 4.1 (SD 1.0) for both arms. We found no significant difference in Quality of Life between the two arms at the primary endpoint (baseline-adjusted difference 0.07, 95% CI −0.07 to 0.21, p = 0.35). The incremental cost-effectiveness ratio (£110,501 per quality-adjusted life-year (QALY)) exceeded the prospectively defined cost-effectiveness threshold (£30,000 per QALY). 158 (42.8%) control arm and 194 (52.4%) intervention arm participants accessed narratives outside of the NEON Intervention. There were no related serious adverse events (SAEs). 116 unrelated SAEs were reported by control arm participants, and 107 by intervention arm participants.

**Interpretation:**

Our findings do not indicate NEON Intervention access for all people with psychosis experience. Future research should consider a) evaluation with current mental health services users; b) optimisation to enable users to find hope-promoting narratives.

**Funding:**

10.13039/501100000272National Institute for Health and Care Research (NIHR).

**Research in context**

**Evidence before this study**

We conducted a systematic review of empirical studies on the impact of mental health recovery narratives on recipients (https://doi.org/10.1177/706743719846108). This included papers published before 30^th^ August 2018, identified from 9 publications databases. Searches included terms loosely synonymous for recovery narrative (e.g. “memoirs”, “autobiographies”), and terms describing ways in which people engage with narratives (e.g. “hear”, “listen”, “read”). Five articles were included. Forms and moderators of impact were identified. We then conducted semi-structured interviews with adults with experience of mental health problems and recovery (n = 77) (https://doi.org/10.1371/journal.pone.0226201). Participants were asked to share a mental health recovery narrative and to describe the impact of other people’s recovery narratives on their own recovery. A preliminary recovery narrative change model was generated through iterative thematic analysis of transcripts. We concluded work on the impact of recovery narratives with an experimental study in which 40 mental health services users were sequentially shown up to 10 recovery narratives, rated their immediate impact, and were interviewed on impact processes (https://doi.org/10.1080/09638237.2021.2022627, https://doi.org/10.1186/s12888-019-2405-z). Evidence across all studies was then synthesized to generate a theory of change for accessing recovery narratives through the NEON Intervention (https://doi.org/10.2196/24417). We have reported findings of the NEON-O Trial for people with mental health problems and no pschysosis experience (https://doi.org/10.1002/Fwps.21176). We found a significant difference in quality of life at 52-week follow-up when comparing the intervention arm to the control arm (Manchester Short Assessment, baseline-adjusted difference 0.13, 95% CI 0.01–0.26, p = 0.041). The Incremental Cost-Effectiveness Ratio (ICER) of £12,526 per Quality Adjusted Life Years compared with usual care was less than a £30,000 cost-effectiveness threshold.

**Added value of this study**

This is the first RCT evaluating the benefits of mental health recovery narratives for people with mental health problems and psychosis experience. We found no evidence for a difference in quality of life between the intervention and control arms (Manchester Short Assessment, baseline-adjusted difference 0.07, 95% CI −0.07 to 0.21, p = 0.35). The ICER (£110,501) exceeded our £30,000 cost-effectiveness threshold, and was only close to the threshold for the participant subgroup who were current users of specialist care mental health services for psychosis (£35,013). In a hypothesis-generating ad hoc analysis, intervention arm users who found at least one narrative hope-promoting experienced a significant increase in quality of life and a significant reduction in distress when compared with the control arm.

**Implications of all the available evidence**

NEON Intervention access is not currently indicated for people with mental health problems and psychosis experience, but should be provided at a population level to people with mental health problems and no psychosis experience. Future research should consider NEON Intervention refinement and evaluation with current users of mental health services for psychosis, and optimisation to facilitate users in finding hope-promoting narratives.

## Introduction

Mental health conditions account for 15.5% of Years Lived with Disability (YLD) globally,^1^ and are largely undertreated. 71% of people with psychosis do not receive specialist mental health services,^1^ with an estimated life expectancy reduction of 10–20 years compared to the general population.^2^ Demand for mental health treatment is rising; mental health services in England received a record 5 million referrals in 2023, an increase in 33% from 2019.^3^ The 2022 WHO *World Mental Health Report* identified that treatment demand is out-stripping supply, and that ‘*business as usual for mental health simply will not do*’.^1^ Innovation is needed. Mental health systems are increasingly organised around a new guiding principle of recovery, no longer prioritising the idea or goal of the person “being cured” but instead focussing on the goal of supporting people to live well in inclusive communities.^4^

Placing increased importance on personal (or lived) experience is central to a recovery orientation. For example, mental health peer support workers are individuals with personal experience of mental health problems, who are employed in mental health services, and who can provide a credible role model of recovery. The peer support work evidence base is robust; an umbrella review concluded on the basis of 426 primary studies that peer support work supports improvements in depression symptoms, self-efficacy, and recovery.^5^ Similarly, Recovery Colleges are an innovative approach involving peer trainers who support recovery through education, with 221 colleges operating in 28 countries.^6^ A common feature of these two initiatives is the involvement of peers willing to share their own narrative of recovery with others. Recovery narratives are also widely available in published forms. A systematic review concluded that published recovery narratives are widely used as a mental health intervention component in healthcare and community settings.^7^ Interventional uses of narratives describing recovery from other health conditions are being explored, including alcohol use disorder.^8^

Through the Narrative Experiences Online (NEON) programme we explored whether mental health recovery narratives can help people affected by mental health problems, and assembled a diverse collection of recovery narratives. We developed and evaluated the NEON Intervention,^9^ a web-based digital health intervention (DHI) providing self-guided and recommender systems access to this collection.^10^ In the NEON-O Trial, we evaluated effectiveness and cost-effectiveness for adults in England experiencing mental health problems, but with no psychosis experience in the previous five years (n = 1023). At the 52-week primary endpoint, we found a significant increase in the mean item score of the Manchester Short Assessment of Quality of Life (MANSA) (baseline adjusted difference 0.13, 95% CI: 0.01–0.26, p = 0.041), and a significant increase in the presence subscale of the Meaning in Life Questionnaire (baseline adjusted difference 0.22, 95% CI 0.05–0.40, p = 0.014). The NEON Intervention was cost-effective from the perspective of the National Health Service (NHS) in England, reducing resource usage for the subgroup of participants who had used specialist mental health services. Population-level implementation is indicated.^11^

Whilst the NEON-O Trial excluded people with psychosis experience, DHIs for psychosis have the potential to improve outcomes,^12^ and with wider access to the NEON Intervention for people without psychosis experience, healthcare professionals will need to decide on clinical relevance for people with psychosis experience. Psychosis experience (rather than current psychosis) is a relevant factor, for example because delusions can persist at a sub-clinical level outside of psychotic episodes,^13^ and may then influence perceptions and hence impact of digital media. We report the definitive NEON Trial for adults in England who have experienced psychosis.

The aim of the NEON Trial was to understand whether access to online recovery narratives benefits people with experience of psychosis. The primary objective was to evaluate the effectiveness of the NEON Intervention in improving quality of life. Secondary objectives were: a) to evaluate effectiveness in improving hope, empowerment and meaning in life, and in reducing psychological distress; b) to assess cost-effectiveness from a health and social care provider perspective; c) to determine whether effectiveness and cost-effectiveness varied according to prior health service usage; d) to understand how the intervention was used. All objectives were evaluated at 52-week follow-up, controlling for usual care.

## Methods

### Trial design and reporting

We conducted a pragmatic, parallel group RCT across England of the NEON Intervention, approved by Leicester Central research ethics committee (19/EM/0326). With sponsor approval, we followed Standard Operating Procedures defined by the Pragmatic Clinical Trials Unit at Queen Mary University of London, who supervised the trial, and employed the Senior Statistician. Oversight was by an independent Programme Steering Committee (PSC). The trial protocol,^14^ Statistical Analysis Plan (SAP),^15^ and baseline characteristics^16^ have been described. The NEON Intervention and all trial procedures were delivered through a web-application verified through a feasibility study^9^ and an internal pilot. Internal pilot data was carried through into the primary trial analysis. Reporting follows the Consolidated Standards of Reporting Trials (CONSORT) 2010^17^ and the Consolidated Health Economic Evaluation Reporting Standards (CHEERS) 2022^18^ statements (Appendices 1 and 2).

### Participants

Inclusion criteria were self-reported: experience of psychosis in the last five years, experience of mental health-related distress in the previous six months, resident in England, aged 18 years or older, capable of accessing or being supported to access the Internet, able to understand written and spoken English, and capable of providing online informed consent. We recruited people with diverse histories of self-reported mental health service use, including none. Participants were recruited by clinical support officers at 11 secondary care research sites, and publicly through community engagement and social media activities.^16^ A planned six-month recruitment evaluation against stop-go rules was not conducted as PSC decided that recruitment at four months was satisfactory. No protocol amendment was required as our protocol allocated PSC the authority to make this decision.

### Randomisation and masking

Participants were randomly assigned to receive immediate (intervention arm) or 52-week delayed (control arm) NEON Intervention access (1:1 allocation ratio, no stratification, permuted blocks with randomly varying block length of 2,4, or 6). All continued with usual care. The automated randomisation system embedded in our web-application was approved by the supervising trials unit. A randomisation list was generated by an independent statistician using the Stata RALLOC package and was stored inaccessibly to the study team. Entries were sequentially used to determine participant allocation status. The trial statistician was masked to treatment allocation until the SAP was approved on 7th December 2021 by the chief investigator, senior statistician, and a PSC-allocated independent statistician. The chief investigator and senior statistician remained masked until trial analysis work was complete. Participants were not masked.

### Procedures

Recruitment work directed potential participants to a website where eligibility was evaluated through a questionnaire, trial information was provided, and informed consent was collected. Participants clicked a link in an automated email to create a password-protected account. A telephone number could be supplied to enable engagement messaging. Sociodemographic and outcome data was collected through web-based questionnaires, with prompts at next login and by email. Participants were randomised after completing baseline questionnaires. Reminders for primary endpoint questionnaires were sent by email, text and phone call (protocol: Appendix 3). A £20 voucher was offered for each set of questionnaires completed. Lateness intervals were specified in the protocol. Our protocol allowed changes to if reasons were documented in the SAP. The 52-week lateness window was adjusted to 91 days due to reports of post-pandemic changes disrupting questionnaire completion. Control arm users gained NEON Intervention access after completing primary endpoint questionnaires.

The NEON Intervention is a web-application providing access to the NEON Collection of recovery narratives (full description: Appendix 4). At first access, participants completed an updatable personal profile defining narrative content to avoid (e.g. text, self-harm, violence). Narratives containing this content were hidden. Participants were then shown a first narrative identified empirically as being hope-promoting.^9^ After receiving this and all subsequent narratives, participants rated immediate impact through validated narrative feedback questions. The first (mandatory) question asked “How hopeful did the story leave you feeling?“. After responding, participants received access to a homepage presenting narrative access mechanisms: (1) Match me to a story; (2) Get me a random story; (3) Browse stories; (4) My Stories. *Match me to a story* selects a narrative not previously accessed, by invoking an automated recommender system. *Get me a random story* selects a narrative not previously accessed, using a random number generator. *Browse stories* allows the selection of a narrative using demographic and content categories. *My stories* allowed return access to narratives previously rated as hope-inspiring or bookmarked by the participant. Content warnings were displayed if relevant. Individual narratives could be hidden.

The homepage also linked to signposting and self-help information. Control arm participants initially received a homepage that retained this information, but excluded narrative access mechanisms until primary endpoint questionnaires were completed. All NEON Intervention usage was logged. The trial opened with 348 narratives, and (per protocol) narratives were added, with 659 available when the final participant reached the primary endpoint. Narratives were not limited to those with psychosis content. Participants were asked to use the NEON Intervention as much or as little as they wished. Engagement messages were sent by email and Short Messaging Service (SMS) (messages: Appendix 5).

Repeat registrations by the same person are an issue in online health studies.^19^ Our protocol allowed suspension of accounts created due to repeat registration. A procedure was developed to manage repeat registration accounts.^20^ In most cases, all accounts associated with repeat registrations were suspended. The account allocated to the intervention arm was retained only when a participant had registered multiple times to gain intervention access, and a clinical risk was determined from study records.

### Outcomes

The primary outcome was quality of life, assessed using the 12 subjective items in MANSA section 3.^21^ This was collected at baseline, week 1, week 12, and (primary endpoint) 52 weeks. Five secondary outcomes were assessed at baseline and 52 weeks. *Psychological distress* was assessed using the Clinical Outcomes in Routine Evaluation-10 (CORE-10).^22^ *Hope* was assessed using the Herth Hope Index.^23^ *Self-efficacy* was assessed through the Mental Health Confidence Scale.^24^ The *presence* and *search* for meaning in life were assessed through the Meaning in Life Questionnaire.^25^ For the economic analysis, *health status* was assessed through the EQ-5D-5L.^26^^,^^27^ An abridged Client Service Receipt Inventory (CSRI) captured health service use data.^28^ Self-reports of post-randomisation recovery narrative usage outside of the NEON Intervention were collected at week 1, week 12 and week 52. Collection forms, psychometric properties and calculation details have been described.^16^

Two web-based forms were provided to allow possible serious adverse events (SAEs) to be reported on occurrence, one accessible to participants who had logged into their account, and one enabling anonymous reporting. Hospitalisations as a form of SAE were also identified retrospectively from the primary endpoint CSRI form. SAEs could be double counted, i.e. reported both on occurrence and through the primary endpoint CSRI form. We did not attempt to identify instances of double counting due to ambiguity, e.g. a participant might enter a different date when initially reporting an SAE and in the CSRI form. In all cases, reports detailing possible SAEs were examined, categorised and actioned by the chief investigator, with participant follow-up if necessary. All SAEs were also reported to PSC annually. SAEs related to trial participation were reported to PSC on occurrence.

### Statistical analysis

The NEON Trial was prospectively registered (ISRCTN11152837, 13th August 2018). It ran in parallel to the NEON-O Trial (ISRCTN63197153)^11^ and the NEON-C Trial (ISRCTN76355273).^29^ The statistical significance level was 5%. Analysis was by a prospectively planned modified Intention To Treat (ITT) principle which excluded accounts suspended due to repeat registration.^15^ There was no interim analysis of outcome data. The economic analysis used Stata version 16.1 (StataCorp LLC). All other analyses used R 64 version 4.1.2 (R Foundation). The primary endpoint was a minimally clinically important difference (MCID) in the mean MANSA item score, defined as an improvement of 0.25 at 52-week follow-up in the intervention arm compared to the control arm.^30^ Allowing for 20% attrition, we prospectively selected a target sample of 684 to provide 90% power [SD = 0.9, power = 0.9, p = 0.05], and an equal number of participants in both arms. The target analysable sample was 546.^14^

### Descriptive analyses

Participant flow was summarised in a prospectively planned CONSORT diagram, adding information on (a) declined consent mechanisms specific to online trials; (b) accounts suspended due to repeat registrations; (c) numbers of participants who received the intervention, defined as receiving at least the introductory recovery narrative. Baseline sociodemographic, clinical, and service use data were summarised by treatment arm. Ethnicity data was reported through pre-defined categories (White, Mixed/Multiple Ethnic background, Asian, Black/African/Caribbean, Arab, Any other ethnic group). The *white* category incorporated minoritised ethnicities, hence the smaller number of participants identifying as *white British* (the majority population) was also summarised. Service use categories were defined in our baseline data analysis.^16^ The Kruskal–Wallis test was used to examine associations between NEON Intervention recovery narrative use and self-reported access to recovery narratives outside of the NEON Intervention (categories: 0, 1–10, 11 or more).

### COVID-19 analysis

Baseline data were collected during a period with restrictions on movement and socialisation due to the COVID-19 pandemic, with repeated periods of national lockdown that imposed severe restrictions. t-tests were used to compare baseline clinical outcome data collected during national lockdown periods with data collected outside of lockdown periods. With MANSA data collected at week 1, week 12 and week 52, a mixed effect model using random effects for intercept parameters and days of measurement from baseline was fitted, and adopted to examine interactions with periods coded as within national lockdown. Lockdown dates are documented in the statistical analysis plan.^15^

### Clinical outcomes analysis

The primary analysis was a linear regression model of outcome at 52-week follow-up, adjusting for baseline score. Multiple Imputation by Chained Equations (MICE)^31^ was performed to impute clinical outcomes and missing baseline predictors of the clinical outcomes in the model using the MI package, with an assumption of Missing at Random (MAR). Fifty datasets were generated, and analyses were combined using Rubin's rules. To explore differential effectiveness, the primary analysis was repeated to include an interaction term between treatment and four baseline participant characteristics: gender, ethnicity, lifetime use of specialist mental health services, and current use of specialist mental health services. Gender and ethnicity were included because the programme theory for our intervention positions connection to the narrator or their narrative as a fundamental part of causal chain by which change is created for the recipient,^9^ with the potential for people with some genders or ethnicities to be advantaged or disadvantaged, for example through the presence or absence of narratives with matching characteristics. Service use items were included due to significant differences in baseline characteristics of service users and nonservice users,^16^ and because service history may be a prognostic factor for outcome in evaluations of mental health DHIs.^16^ We explored the sensitivity of findings to protocol deviations through complete case and per-protocol analyses. We explored sensitivity to missingness through a complete case analysis with significant predictors of missingness added as covariates. Ad hoc analyses were conducted using baseline-adjusted linear regressions to compare clinical outcomes for intervention arm sub-groups including participants who a) received the intervention (e.g. accessed at least one narrative); b) rated at least one narrative as much more hopeful. The comparison was the control group. These ad hoc analyses should be considered hypothesis-generating only.

### Health economic analysis

The cost-effectiveness analysis compared the cost and quality-adjusted life-years (QALYs) gained for both arms from the perspective of the NHS in England. All costs are reported in Pounds Sterling (£) (price year: 2020/21). The time horizon was 12 months, so costs and QALYs were not discounted. The per-participant delivery cost (£321) comprised the total trial delivery cost divided by the number of intervention arm participants.^32^ TAU was assumed to have zero upfront cost because the NEON Intervention was an addition to participants' current levels of care. Downstream healthcare resource use was calculated for both arms using the CSRI and combined with UK-based unit costs (Table S1, Appendix 6). EQ-5D-5L data collected at baseline and 52-week follow-up were used to estimate health status. EQ-5D-5L responses were converted to EQ-5D-3L utility values (UK tariff), following the National Institute for Health and Care Excellence's reference case.^33^ The mapping method^34^ required a binary model for participant sex, whereas we collected gender using a nonbinary model. As sex and gender are separate constructs, EQ-5D-3L values were treated as missing for participants responding *other* for gender. QALYs were calculated from the utility values for each participant, assuming a linear relationship between the time points.^35^

Mean total cost (log-link and Gamma family) and QALYs (identity-link and Gaussian family) were estimated for each arm using generalised linear models and recycled predictions adjusting for trial allocation and baseline characteristics (age, gender, MANSA score), baseline EQ-5D-3L utility value, and baseline cost (cost regression only).^35^ Missing data were handled with MICE assuming MAR. The main outcome was the incremental cost-effectiveness ratio (ICER) (Equation (1)).

(1) $$\begin{array}{c} ICER=\frac{Cost\left(Intervention\right)\;-\;Cost\left(Control\right)}{QALY\left(Intervention\right)\;-\;QALY\left(Control\right)} \end{array}$$

Uncertainty was handled by bootstrapping (n = 2000 replications). 95% credible intervals report the distribution in which 95% of replications occurred. Cost-effectiveness was determined against a threshold of £30,000 per QALY gained.^33^ For the base-case analysis, the joint distribution of incremental costs and QALYs was illustrated on a cost-effectiveness plane and the probability of cost-effectiveness was illustrated using a cost-effectiveness acceptability curve. Sensitivity analyses were performed to assess the robustness of the base case results under different analysis assumptions (Table S2, Appendix 6). The analysis was repeated to examine subgroups containing lifetime specialist service users and current specialist service users.

### Role of the funding source

The funders and sponsor had no role in the design of the study; in the collection, analysis, and interpretation of data; in the writing of the report; and in the decision to submit the paper for publication.

## Results

**Fig. 1:**
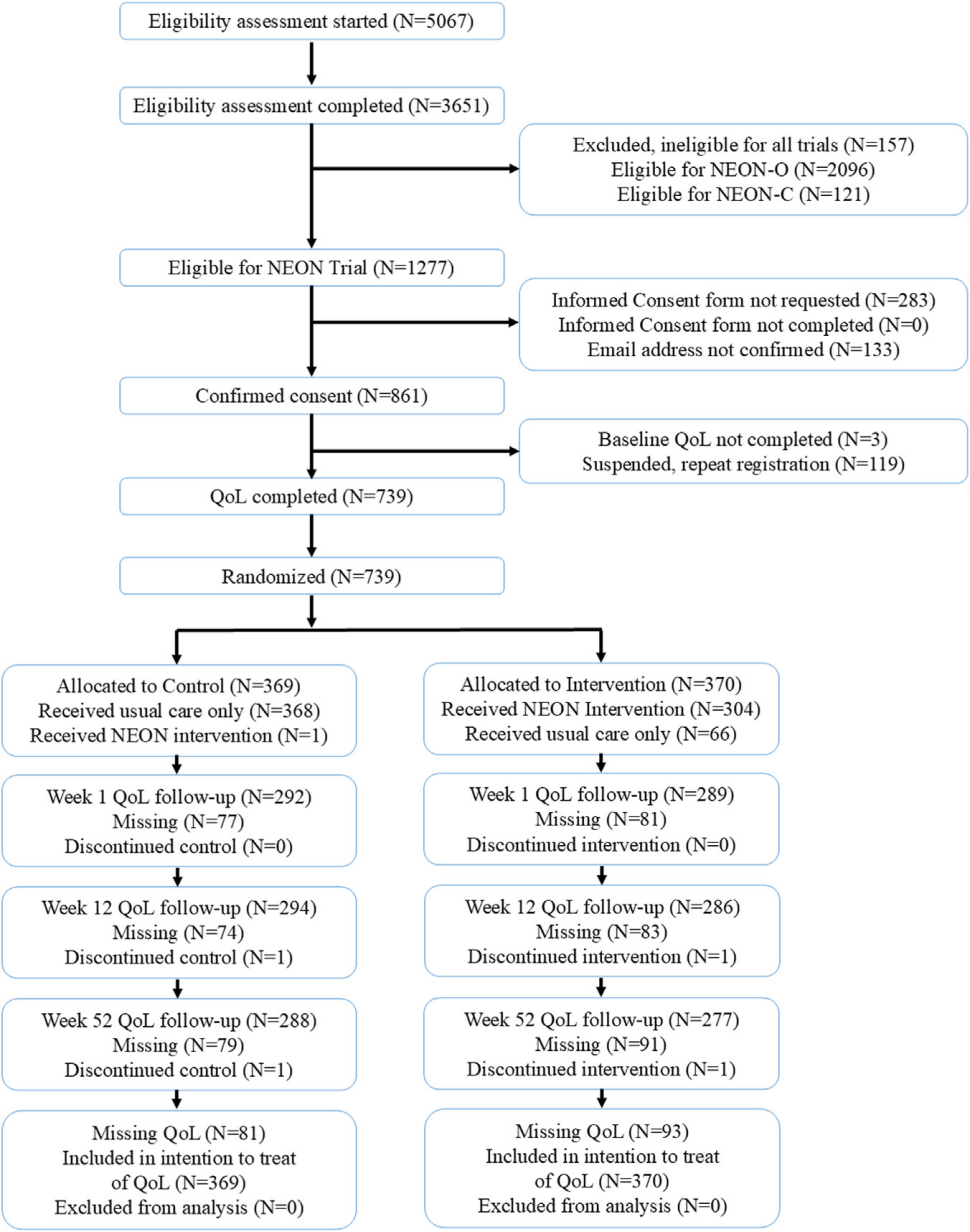
CONSORT diagram. QoL: Quality of Life. Suspensions through the repeat registration protocol could occur at any point from consent form completion onwards, but are included as a single entry for clarity of reporting.

Recruitment opened on 9 March 2020. The first and last completed baseline assessments were 10 March 2020 and 13 February 2021 respectively. The web-application was closed to new eligibility assessments on 1 March 2021. The final item of primary outcome data was collected on 9 March 2022. The trial was closed to follow-up data on 22 September 2022, at which point our web-based logging system was locked to further data collection, and files containing all logged data were downloaded to our analysis server and marked as read-only. The modified ITT sample (n = 739) was balanced between the arms (intervention: n = 370; control: n = 369) and larger than the target (n = 684) due to planned over-recruitment authorised by the study sponsor, with prior PSC approval. One control arm participant received early intervention access due to a technology error. Once identified, access was suspended until after 52-week follow-up. Fig. 1 shows participant flow.

Baseline sociodemographic items are summarised in Table 1.

**Table 1:** Baseline demographic items collected through the online demographics form.

	Control N = 369^d^	Intervention N = 370^d^	Total N = 739^d^
**Gender, n (%)**			
Female	235 (63.7)	208 (56.2)	443 (59.9)
Male	123 (33.3)	151 (40.8)	274 (37.1)
Other	6 (1.6)	10 (2.7)	16 (2.2)
**Age (years)** mean (SD)	35.2 (12.2)	34.5 (11.8)	34.8 (12)
**Ethnicity, n (%)**			
White	317 (85.9)	321 (86.8)	638 (86.3)
White British^a^	283 (76.7)	278 (75.1)	561 (75.9)
Mixed/Multiple Ethnic background	19 (5.1)	21 (5.7)	40 (5.4)
Asian	15 (4.1)	18 (4.9)	33 (4.5)
Back/African/Caribbean	11 (3.0)	5 (1.4)	16 (2.2)
Arab *or* Any other ethnic group^b^	Redacted^c^	Redacted^c^	6 (0.8)
**Region of current residence, n (%)**			
East of England	30 (8.1)	23 (6.2)	53 (7.2)
London	84 (22.8)	82 (22.2)	166 (22.5)
Midlands	54 (14.6)	58 (15.7)	112 (15.2)
North East and Yorkshire	37 (10.0)	43 (11.6)	80 (10.8)
North West	32 (8.7)	34 (9.2)	66 (8.9)
South East	67 (18.2)	66 (17.8)	133 (18)
South West	60 (16.3)	63 (17)	123 (16.6)
**Education, highest qualification, n (%)**			
No qualification	25 (6.8)	26 (7.0)	51 (6.9)
O-levels/GCSE	58 (15.7)	59 (15.9)	117 (15.8)
A-levels/AS-levels/NVQ or equivalent	140 (37.9)	138 (37.3)	278 (37.6)
Degree-level qualification	98 (26.6)	109 (29.5)	207 (28.0)
Higher degree level qualification	43 (11.7)	37 (10.0)	80 (10.8)
**Best description of recovery, n (%)**			
I don't want to say	20 (5.4)	28 (7.6)	48 (6.5)
Not yet thinking about recovery	45 (12.2)	46 (12.4)	91 (12.3)
Working on recovery	251 (68.0)	259 (70.0)	510 (69.0)
Living beyond disability	48 (13.0)	36 (9.7)	84 (11.4)
**Mental health service use**			
Have ever used primary care mental health services	344 (93.2)	354 (95.7)	698 (94.5)
Have ever used specialist care mental health services	300 (81.3)	309 (83.5)	609 (82.4)
Current user of specialist care mental health services	137 (37.1)	142 (38.4)	279 (37.7)
**Main Mental Health Problem in last month, n (%)**			
I don't want to say	7 (1.9)	13 (3.5)	20 (2.7)
I did not experience mental health problems	9 (2.4)	10 (2.7)	19 (2.6)
Developmental disorder such as learning disability	10 (2.7)	5 (1.4)	15 (2.0)
Eating disorder such as anorexia or bulimia	8 (2.2)	7 (1.9)	15 (2.0)
Mood disorder such as depression, anxiety or bipolar	134 (36.3)	131 (35.4)	265 (35.9)
Personality disorder such as borderline personality disorder	66 (17.9)	72 (19.5)	138 (18.7)
Schizophrenia or other psychosis such as schizo-affective disorder or delusional disorder	74 (20.1)	80 (21.6)	154 (20.8)
Stress-related disorders such as PTSD or OCD	40 (10.8)	42 (11.4)	82 (11.1)
Substance-related disorder such as alcohol or drug misuse	16 (4.3)	9 (2.4)	25 (3.4)
**Residential status, n (%)**			
Alone	96 (26.0)	119 (32.2)	215 (29.1)
With Others	273 (74.0)	251 (67.8)	524 (70.9)
**Occupation, n (%)**			
Employed	142 (38.5)	135 (36.5)	277 (37.5)
Sheltered employment	5 (1.4)	5 (1.4)	10 (1.4)
Training and education	35 (9.5)	41 (11.1)	76 (10.3)
Unemployed	177 (48.0)	179 (48.4)	356 (48.2)
Retired	10 (2.7)	10 (2.7)	20 (2.7)

a Compared to the white category, the white British category excludes people with white minoritised ethnicities.

b Rows combined.

c Finding redacted due to less than 5 participants.

d N = 369; N = 370; N = 739: these refer to the number of participants in the control arm, intervention arm, and trial sample respectively. For most items, data was missing for 5 control arm participants and 1 intervention arm participant. The exception was the items on *residential status* and *occupation*, where no data was missing.

Baseline clinical characteristics are summarised in Table 2.

**Table 2:** Baseline clinical outcomes.

	Control (N = 369)	Intervention (N = 370)	Total (N = 739)
**MANSA** Mean (SD)	3.7 (0.9)	3.7 (1.0)	3.7 (0.9)
Missing, n (%)	0 (0.0)	0 (0.0)	0 (0.0)
**CORE-10** Mean (SD)	22.5 (7.5)	23 (7.2)	22.7 (7.3)
Missing, n (%)	10 (2.7)	3 (0.8)	13 (1.8)
**Herth Hope Index** Mean (SD)	28.8 (6.8)	28.5 (6.8)	28.7 (6.8)
Missing, n (%)	10 (2.7)	3 (0.8)	13 (1.8)
**Mental Health Confidence Scale** Mean (SD)	50.5 (14.0)	49.4 (14.5)	49.9 (14.2)
Missing, n (%)	10 (2.7)	5 (1.4)	15 (2.0)
**Meaning in life: Presence subscale** Mean (SD)	3.5 (1.4)	3.4 (1.4)	3.5 (1.4)
**Meaning in life: Search subscale** Mean (SD)	4.6 (1.4)	4.6 (1.4)	4.6 (1.4)
Missing, n (%)	10 (2.7)	6 (1.6)	16 (2.2)
**EQ-5D-3L** Median (IQR)	0.5 (0.3–0.7)	0.5 (0.3–0.7)	0.5 (0.3–0.7)
Missing, n (%)	10 (2.7)	6 (1.6)	16 (2.2)

Ranges for outcomes are MANSA [1 to 7]; CORE-10 [0 to 40]; Herth Hope Index [12 to 48]; Mental Health Confidence Scale [16 to 96]; Meaning in Life (presence and search subscales) [1 to 7]; EQ-5D-3L [−0.6 to 1].

In this baseline data, mean age was 34.8 (SD 12.0), 443 (59.9%) identified as female, and 561 (75.9%) as White British. 261 (35.3%) had been in contact with a specialist community mental health team for their psychosis experience, and 18 (2.4%) were in patients. 100 (13.5%) had had no contact with any NHS service for their psychosis experience. For their primary mental health problem, 265 (35.9%) identified mood disorders (which included bipolar disorder) as their main mental health problem in the last month, whilst 154 (20.8%) identified schizophrenia as their main mental health problem in the last month.

### Effectiveness analyses

565 (76.5%) participants completed the primary endpoint MANSA questionnaire, greater than the planned analysable sample of 546. The mean primary endpoint MANSA score was 4.07 (SD 1.0) for the control arm and 4.13 (SD 1.0) for the intervention arm. Highest educational qualification at baseline (p = 0.0005), hope at baseline (p = 0.008) and mental health confidence at baseline (p = 0.038) predicted missingness in the primary endpoint MANSA score, but did not predict the score itself, supporting the assumption of MAR. Predictors of missingness for all outcomes are in Table S3 (Appendix 7). For the multiple imputation model, the best predictors of the MANSA week 52 score were the MANSA week 1 and the MANSA week 12 score. Including imputed data for the primary analysis, we found no significant difference in quality of life between treatment arms (baseline-adjusted difference 0.07, 95% CI −0.07 to 0.21, p = 0.35), and no significant difference for any secondary outcome. Findings are in Table 3.

**Table 3:** Primary analysis of effectiveness.

	Baseline-adjusted difference (95% CI)	p-value
MANSA	0.07 (−0.07 to 0.21)	0.35
CORE-10	0.01 (−1.06 to 1.26)	0.99
Herth Hope Index	−0.47 (−1.46 to 0.53)	0.36
Mental Health Confidence Scale	0.10 (−2.20 to 2.41)	0.93
Meaning in life: Presence subscale	−0.09 (−0.29 to 0.11)	0.39
Meaning in life: Search subscale	−0.01 (−0.18 to 0.20)	0.90

Analysis of the ITT sample (control n = 369; intervention n = 370) with missing data imputed. Ranges for outcomes are MANSA [1 to 7]; CORE-10 [0 to 40]; Herth Hope Index [12 to 48]; Mental Health Confidence Scale [16 to 96]; Meaning in Life (presence and search subscales) [1 to 7]; EQ-5D-3L [−0.59 to 1].

There were no significant differences for the complete case analysis (Table S4, Appendix 7). The MANSA finding was not sensitive to protocol deviations (Table S5, Appendix 7) or to missingness (Table S6, Appendix 7). There were no significant interaction effects with gender, ethnicity, or service use history (Table S7, Appendix 7). There were no SAEs related to the NEON Intervention or trial procedures. 116 unrelated SAEs were reported by control arm participants, and 107 unrelated SAEs were reported by intervention arm participants. Of these 223 unrelated SAEs, 213 were reported through the primary endpoint CSRI form and 10 through our web-based safety reporting forms.

In our ad hoc analyses, no significant differences in clinical outcome were found when comparing the 304 (82.2%) participants who received the intervention, with the control arm. When comparing intervention arm participants who had rated at least one narrative as “much more hopeful” with control arm participants, there was a significant baseline-adjusted increase in the MANSA score that was above the MCID (0.34, 95% CI 0.12–0.56, p = 0.0026). There was a significant baseline-adjusted decrease in CORE-10 (−2.69, 95% CI −4.45 to −0.92, p = 0.0029) (Table S8, Appendix 7).

When we conducted t-tests, there was no evidence that national lockdown influenced baseline clinical characteristics (Table S9, Appendix 7). Our mixed-effects model (intercept and time (in days) as random effects, randomisation, time (in days), MANSA baseline and lockdown yes/no as fixed effect) incorporated MANSA data from all timepoints (week 1, week 12, week 52, 689 participants, 1718 observations). It found no evidence that national lockdown influenced MANSA follow-up data (β = −0.6, standard error = 0.04, p = 0.18).

### Cost-effectiveness

Total cost data were available for 70.5% of participants in the intervention arm and 80.0% in the control arm. Total QALY data were available for 67.6% in the intervention arm and 74.0% in the control arm. Missing data are summarised in Table S10, Appendix 7.

Table 4 summarises base-case findings. In the adjusted base-case analysis, which was the main result, the estimated ICER was £110,501 per QALY gained, which exceeded the threshold to determine cost-effectiveness in England.

**Table 4:** Base case economic analysis.

Analysis	Cost			QALYs			ICER
	Intervention	Control	Incremental	Intervention	Control	Incremental	
Adjusted base case	£3465	£2288	£1177 (£438–£1969)	0.5261	0.5154	0.0107 (−0.0041 to 0.0258)	£110,501
Unadjusted base case	£4548	£2835	£1712 (£569–£2990)	0.5189	0.5226	−0.0036 (−0.0399 to 0.0343)	Dominated

Analysis of the ITT sample (control n = 369; intervention n = 370) with missing data imputed. Incremental results compare Intervention to Control (95% Bayesian credible interval for incremental outcomes reported in parentheses). ICER: Incremental cost-effectiveness ratio. QALY: Quality-adjusted life year.

There was no uncertainty that the NEON Intervention was cost-increasing but uncertainty over the direction of QALY gain, which is illustrated in the cost-effectiveness plane in Figure S4 (Appendix 7). At a threshold of £30,000 per QALY gained, the probability that the NEON Intervention was cost-effective was 3.9% which is illustrated in Figure S5 (Appendix 7).

The adjusted base-case was robust to all sensitivity analyses (Table S11, Appendix 7). When the costs of delivering the NEON Intervention were omitted (sensitivity analysis D), the incremental cost was £720 (95% credible interval -£30 to £1494) indicating that, on average, downstream health service resource use increased in the intervention arm compared with the control arm. The cost-effectiveness of the NEON Intervention did not change for subgroups containing participants who at baseline had ever used specialist mental health services, or who were currently using specialist mental health services (Table S12, Appendix 7).

### Engagement

For intervention arm participants who received the intervention, the median number of narrative requests was 4 (IQR: 2–11, minimum: 1, maximum: 205). In total, 231 (76.0%) of these participants provided at least one narrative feedback item. Of the 3254 intervention-arm narrative requests, 2028 (62.3%) requests received a feedback item on hope. 1038 (53.4%) indicated increased hope and 178 (8.8%) decreased hope. The distribution is in Table S13 (Appendix 7).

### Non-NEON narrative usage

At 52-week follow-up, 158 (42.8%) control arm participants and 194 (52.4%) intervention arm participants had accessed recovery narratives outside of the NEON Intervention. Descriptive statistics are in Table S14 (Appendix 7). By 12-week and 52-week follow-up, those who had accessed more recovery narratives through the NEON Intervention had also reported accessing more public recovery narratives (Kruskal–Wallis test: p < 0.0001 for each), but there was no difference by week 1 (p = 0.060).

## Discussion

We conducted a prospectively registered RCT of the NEON Intervention. We met our targets for initial recruitment and the size of the 52-week analysable sample. There was no significant change in the primary outcome or any secondary outcome. NEON Intervention access increased costs from the perspective of the NHS in England. It increased QALYs by 0.0107 per participant, but the 95% credible interval crossed 0, the ICER was substantially above the selected threshold for cost-effectiveness, and hence the NEON Intervention was not cost-effective for our population. From the available evidence, NEON Intervention access is not indicated for adults with psychosis experience. There were no related serious adverse events, and hence no immediate evidence for harm from intervention access.

The generalisability of our findings is limited by an unrepresentative proportion of female participants, and whilst we examined differential effects by gender, it is possible that the small proportion of male participants obscured a differential effect. No information on psychosis characteristics or duration was captured. Our economic analysis does not generalise to participants not identifying as female or male. Recovery narratives were presented exactly as created by their narrators. This was strength because it avoided a risk of harm to the narrator by inadvertently changing a meaningful narrative component. It was a weakness because it created accessibility limitations; for example, narratives presented as images were inaccessible to people with visual impairments. We cannot exclude the possibility that more than one person could have shared a NEON account (and hence used the NEON Intervention or completed online measures questionnaires), as we chose not to use potentially intrusive identify verification procedures. Our approach to safety data collection was limited as we only monitored SAEs. Since important safety concerns can be identified through the inspection of non-serious adverse events,^36^ we cannot draw a definitive conclusion on intervention safety. Recovery narratives are now widely available to the public, and whilst public recovery narrative access lacks some possible NEON Intervention benefits (our consistent approach to content warnings; our mechanisms for enabling relevant narratives to be identified), widespread access to public recovery narrative by control arm participants is a source of contamination.

To enable public recruitment, and to reduce barriers to trial participation, psychosis experience was self-assessed, but this risks a range of forms of inaccuracy, including through recall bias around the five-year window we used on psychosis experience. However, misdiagnosis of mental health problems is a widespread phenomenon, and hence there is no “gold standard” for characterising the mental health problems of participants. In our baseline demographics form, we asked about the main mental health problem experienced in the last month, only allowing one option to be selected. Participants were not asked about current psychosis experience; hence we could not define a subgroup of participants still experiencing psychosis.

The parallel NEON-O Trial found a significant improvement in the same primary outcome, despite NEON-O Trial intervention arm participants receiving less recovery narratives than NEON Trial intervention arm participants (median 3 versus 4). This was cost-effective when assessed at the same threshold used in the NEON Trial. Whilst one interpretation is that there was a difference in NEON Intervention effectiveness and cost-effectiveness between the study populations in these two trials, other interpretations are possible. The only logistical difference between the trials is that NEON Trial participants were paid for all questionnaire responses, whilst NEON-O Trial participants were only paid for primary endpoint responses. The proportion of NEON Trial intervention arm participants who received the intervention is lower than for the NEON-O Trial (NEON Trial: 82.2% (304/370), NEON-O Trial: 93.3% (473/507)). Our process evaluation found that some NEON Trial participants reported registering for the purposes of receiving payment vouchers.^37^ These figures are consistent with the NEON Trial recruiting a greater proportion of participants who were motivated to claim voucher payments rather than to engage with the NEON Intervention. This is a possible weakness of the NEON Trial, with the potential to contribute to a null result.

Our primary objective was to evaluate the effectiveness of the NEON Intervention in improving quality of life at 52-week follow-up. There are three plausible reasons that no effect was found in our trial: 1) The NEON Intervention may not be effective at improving quality of life for the chosen population; 2) The NEON Intervention may be effective, this effect is predominantly caused by recovery narrative access (rather than, for example, the online advice that the NEON Intervention provides for people experiencing distress), and the effect was diluted, through either a) the 66 intervention arm participants who accessed no narratives; or b) contamination due to the 158 control arm participants who accessed public recovery narratives. Future research could address this ambiguity.

In an ad hoc analysis, we found a significant baseline-adjusted increase in the MANSA score for intervention arm participants who had rated a narrative as making them feel “much more hopeful”, in comparison to control arm participants. For the same participants, we found a significant reduction in distress. This finding is consistent with a hypothesis that hope-promotion is a mediator in narrative impact. It suggests an approach to the curation of narrative collections that maximises opportunity for hope promotion. This hypothesis and approach might be explored through future research. Alternative interpretations are possible, and should be explored. For example, differences in perception of narrative hope promotion may be due to differences in current mental health symptomatology or demographics.

Whilst the NEON Intervention was not cost-effective for the sample as a whole, or for any prospectively-defined subgroup, the ICER was close to the £30,000 threshold for people who were current users of specialist care mental health services (£35,013, Table S12, Appendix 7). Most health care technologies have lower per-user delivery costs as scale increases,^38^ and hence a lower ICER would be expected in a scenario where the NEON Intervention was deployed on a larger scale. Given that the ICER may also be overstated due to the issues with contamination or no intervention use raised above, then further investigation of NEON Intervention effectiveness and cost-effectiveness for mental health service users is warranted. Clinical focus groups have highlighted opportunities, enablers, and barriers in a clinical context.^39^ A need for further investigation is reinforced by a parallel finding from the NEON-O Trial, where intervention access increased QALYs and reduced NHS resource consumption for people who had used specialist care mental health services at baseline, which was the closest comparable subgroup.

Given that public recovery narratives are now readily available; future evaluations of recovery narrative impact may consider alternative design to trials. For example, a nationally representative survey could examine associations between recovery narrative access and recovery status, or existing cohort studies could be modified to collect relevant data.

## Contributors

The corresponding author attests that all listed authors meet authorship criteria and that no others meeting the criteria have been omitted. Mike Slade, Rachel Elliott, Kristian Pollock, Stefan Priebe, Graham Thornicroft, Julie Repper and Jeroen Keppens secured funding for the NEON study. Mike Slade, Stefan Rennick-Egglestone, Caroline Yeo, Clare Robinson, Chris Newby, Rachel Ellott, Kristian Pollock, Jeroen Keppens, Melanie Smuk, Pim Cuijpers, Rianna Walcott, Joy Llewellyn-Beardsley and Fiona Ng developed and updated the trial protocol. Mike Slade, Stefan Rennick-Egglestone, Clare Robinson, Chris Newby, Rachel Elliott, Melanie Smuk, Joy Llewellyn-Beardsley and Fiona Ng developed the statistical analysis plan. Mike Slade, Stefan Rennick-Egglestone, Rachel Elliott, Sean Gavan and Luke Paterson developed the health economic analysis plan. Mike Slade, Stefan Rennick-Egglestone, Caroline Yeo, Jeroen Keppens, Donna Franklin, Rianna Walcott, Julian Harrison, Dan Robotham, Joy Llewellyn-Beardsley and Fiona Ng developed the NEON Intervention. Tony Glover and Jeroen Keppens produced the source code for the NEON Intervention. Donna Franklin, Rianna Walcott, Julian Harrison, Simon Bradstreet, Steve Gillard, Pim Cuijpers, Marianne Farkas, and Dror Ben-Zeev advised on NEON Intervention safety strategies. Mike Slade, Stefan Rennick-Egglestone, Caroline Yeo, Yasmin Ali, Joy Llewellyn-Beardsley, Fiona Ng, Pim Cuijpers and Dror Ben-Zeev developed NEON Intervention engagement strategies. Mike Slade, Stefan Rennick-Egglestone, Yasmin Ali, Caroline Yeo, Donna Franklin, Julian Harrison, James Roe, Joy Llewellyn-Beardsley and Fiona Ng conducted participant recruitment work. Yasmin Ali, Caroline Yeo, James Roe, Joy Llewellyn-Beardsley and Fiona Ng collected trial data. Chris Newby cleaned trial data and conducted descriptive and clinical outcome analyses. Sean Gavan and Luke Paterson conducted economic analyses. Stefan Rennick-Egglestone, Rachel Elliott and Clare Robinson conducted quality assurance work. Mike Slade, Stefan Rennick-Egglestone and Rachel Elliott drafted initial text for this document. All authors reviewed and critically commented on text. Chris Newby and Stefan Rennick-Egglestone have accessed and verified the data in full. Sean Gavan has accessed and verified data used in the economic analysis. All authors accept responsibility for the decision to submit for publication. All authors had full access to data on request. Mike Slade, Stefan Rennick-Egglestone, Clare Robinson and Rachel Elliott were responsible for the decision to submit the manuscript.

## Data sharing statement

The study sponsor has responsibility for data sharing until the end of the retention period (22 September 2027). To fulfil this responsibility, data will be published through the UK Data Service, or through an equivalent repository if the UK Data Service becomes unavailable. Only anonymous and pseudonymous elements of the datasets used or analysed during the study will be available. Informed consent information has been retained for audit but will not be shared. Some categories of demographic data will be redacted to avoid risk of re-identification. A data dictionary will be provided. The study protocol has been published. The statistical analysis plan has been published. Data access is controlled to protect the confidentiality of trial participants, including to avoid re-identification through combination of multiple data files. An end-user license must be signed by an authorised representative before access can be granted. The end-user license includes a legal commitment to good practices in data processing. Beyond the end of the retention period, we envisage maintaining data availability through a public data repository, but this is at the discretion of the study sponsor. This publication must be cited in all published secondary analyses.

## Declaration of interests

All authors had financial support from the National Institute for Health and Care Research (NIHR) for the submitted work; Graham Thornicroft had financial support from the UK Medical Research Council for the submitted work; Rachel Elliott had financial support from Abbott Diabetes Care, NHS England, CSL Behring, and Fonds voor Wetenschappelijk Onderzoe for the submitted work; Rachel Elliott is a Trusty of Pharmacy Research UK. Other than these declared interests, no author had financial relationships with any organisations that might have an interest in the submitted work in the previous three years; no author had other relationships or activities that could appear to have influenced the submitted work. Intellectual property rights for the NEON Intervention are owned by the study sponsor, Nottinghamshire Healthcare NHS Foundation Trust, who have responsibility for commercial exploitation.
