# Supplementary material for: Effectiveness and cost-effectiveness of online recorded recovery narratives in improving quality of life for people with psychosis experience (NEON Trial): a pragmatic randomised controlled trial
Source: Lancet Reg Health Eur. 2024 Oct 23;47:101101. doi: 10.1016/j.lanepe.2024.101101 (PMC11539663; doi:10.1016/j.lanepe.2024.101101)
Supplement: Multimedia component 1 [file mmc1.docx]

Supplementary information about the NEON Trial

[Appendix 1. CONSORT 2010 checklist. 2](#_Toc177397518)

[Appendix 2. CHEERS checklist. 4](#_Toc177397519)

[Appendix 3. Protocol for primary endpoint outcome data collection. 7](#_Toc177397520)

[Appendix 4. Description of the NEON Collection and the NEON Intervention 12](#_Toc177397521)

[Appendix 5. NEON Intervention engagement messages. 17](#_Toc177397522)

[Appendix 6. Supplementary information about the health economics analysis 32](#_Toc177397523)

[Appendix 7. Supplementary findings from the NEON Trial. 35](#_Toc177397524)

[References for all appendices. 49](#_Toc177397525)

# Appendix 1. CONSORT 2010 checklist.

This appendix uses a checklist published as part of the CONSORT 2010 guidelines.^1^

|  |  | Reporting Item | Page Number |
| --- | --- | --- | --- |
| Title and Abstract |  |  |  |
| Title | [#1a](https://www.goodreports.org/reporting-checklists/consort/info/#1a) | Identification as a randomized trial in the title. | 1 |
| Abstract | [#1b](https://www.goodreports.org/reporting-checklists/consort/info/#1b) | Structured summary of trial design, methods, results, and conclusions | 3 |
| Introduction |  |  |  |
| Background and objectives | [#2a](https://www.goodreports.org/reporting-checklists/consort/info/#2a) | Scientific background and explanation of rationale | 4 |
| Background and objectives | [#2b](https://www.goodreports.org/reporting-checklists/consort/info/#2b) | Specific objectives or hypothesis | 4 |
| Methods |  |  |  |
| Trial design | [#3a](https://www.goodreports.org/reporting-checklists/consort/info/#3a) | Description of trial design (such as parallel, factorial) including allocation ratio. | 5 |
| Trial design | [#3b](https://www.goodreports.org/reporting-checklists/consort/info/#3b) | Important changes to methods after trial commencement (such as eligibility criteria), with reasons | 5 |
| Participants | [#4a](https://www.goodreports.org/reporting-checklists/consort/info/#4a) | Eligibility criteria for participants | 5 |
| Participants | [#4b](https://www.goodreports.org/reporting-checklists/consort/info/#4b) | Settings and locations where the data were collected | 5,6 |
| Interventions | [#5](https://www.goodreports.org/reporting-checklists/consort/info/#5) | The experimental and control interventions for each group with sufficient details to allow replication, including how and when they were actually administered | 5,6 |
| Outcomes | [#6a](https://www.goodreports.org/reporting-checklists/consort/info/#6a) | Completely defined prespecified primary and secondary outcome measures, including how and when they were assessed | 6 |
| Sample size | [#7a](https://www.goodreports.org/reporting-checklists/consort/info/#7a) | How sample size was determined. | 6 |
| Sample size | [#7b](https://www.goodreports.org/reporting-checklists/consort/info/#7b) | When applicable, explanation of any interim analyses and stopping guidelines | 5 |
| Randomization - Sequence generation | [#8a](https://www.goodreports.org/reporting-checklists/consort/info/#8a) | Method used to generate the random allocation sequence. | 5 |
| Randomization - Sequence generation | [#8b](https://www.goodreports.org/reporting-checklists/consort/info/#8b) | Type of randomization; details of any restriction (such as blocking and block size) | 5 |
| Randomization - Allocation concealment mechanism | [#9](https://www.goodreports.org/reporting-checklists/consort/info/#9) | Mechanism used to implement the random allocation sequence (such as sequentially numbered containers), describing any steps taken to conceal the sequence until interventions were assigned | 5 |
| Randomization - Implementation | [#10](https://www.goodreports.org/reporting-checklists/consort/info/#10) | Who generated the allocation sequence, who enrolled participants, and who assigned participants to interventions | 5 |
| Blinding | [#11a](https://www.goodreports.org/reporting-checklists/consort/info/#11a) | If done, who was blinded after assignment to interventions (for example, participants, care providers, those assessing outcomes) and how. | 5 |
| Blinding | [#11b](https://www.goodreports.org/reporting-checklists/consort/info/#11b) | If relevant, description of the similarity of interventions | NA |
| Statistical methods | [#12a](https://www.goodreports.org/reporting-checklists/consort/info/#12a) | Statistical methods used to compare groups for primary and secondary outcomes | 6,7 |
| Statistical methods | [#12b](https://www.goodreports.org/reporting-checklists/consort/info/#12b) | Methods for additional analyses, such as subgroup analyses and adjusted analyses | 6,7 |
| Outcomes | [#6b](https://www.goodreports.org/reporting-checklists/consort/info/#6b) | Any changes to trial outcomes after the trial commenced, with reasons | NA |
| Results |  |  |  |
| Participant flow diagram (strongly recommended) | [#13a](https://www.goodreports.org/reporting-checklists/consort/info/#13a) | For each group, the numbers of participants who were randomly assigned, received intended treatment, and were analysed for the primary outcome | 8 |
| Participant flow | [#13b](https://www.goodreports.org/reporting-checklists/consort/info/#13b) | For each group, losses and exclusions after randomization, together with reason | 8 |
| Recruitment | [#14a](https://www.goodreports.org/reporting-checklists/consort/info/#14a) | Dates defining the periods of recruitment and follow-up | 8 |
| Recruitment | [#14b](https://www.goodreports.org/reporting-checklists/consort/info/#14b) | Why the trial ended or was stopped | 8 |
| Baseline data | [#15](https://www.goodreports.org/reporting-checklists/consort/info/#15) | A table showing baseline demographic and clinical characteristics for each group | 8 |
| Numbers analysed | [#16](https://www.goodreports.org/reporting-checklists/consort/info/#16) | For each group, number of participants (denominator) included in each analysis and whether the analysis was by original assigned groups | 8 |
| Outcomes and estimation | [#17a](https://www.goodreports.org/reporting-checklists/consort/info/#17a) | For each primary and secondary outcome, results for each group, and the estimated effect size and its precision (such as 95% confidence interval) | 8,9 |
| Outcomes and estimation | [#17b](https://www.goodreports.org/reporting-checklists/consort/info/#17b) | For binary outcomes, presentation of both absolute and relative effect sizes is recommended | NA |
| Ancillary analyses | [#18](https://www.goodreports.org/reporting-checklists/consort/info/#18) | Results of any other analyses performed, including subgroup analyses and adjusted analyses, distinguishing pre-specified from exploratory | 8,9 |
| Harms | [#19](https://www.goodreports.org/reporting-checklists/consort/info/#19) | All important harms or unintended effects in each group (For specific guidance see CONSORT for harms) | 9 |
| Discussion |  |  |  |
| Limitations | [#20](https://www.goodreports.org/reporting-checklists/consort/info/#20) | Trial limitations, addressing sources of potential bias, imprecision, and, if relevant, multiplicity of analyses | 10,11 |
| Interpretation | [#22](https://www.goodreports.org/reporting-checklists/consort/info/#22) | Interpretation consistent with results, balancing benefits and harms, and considering other relevant evidence | 10,11 |
| Registration | [#23](https://www.goodreports.org/reporting-checklists/consort/info/#23) | Registration number and name of trial registry | 6 |
| Generalisability | [#21](https://www.goodreports.org/reporting-checklists/consort/info/#21) | Generalisability (external validity, applicability) of the trial findings | 10,11 |
| Other information |  |  |  |
| Interpretation | [#22](https://www.goodreports.org/reporting-checklists/consort/info/#22) | Interpretation consistent with results, balancing benefits and harms, and considering other relevant evidence | 10,11 |
| Registration | [#23](https://www.goodreports.org/reporting-checklists/consort/info/#23) | Registration number and name of trial registry | 6 |
| Protocol | [#24](https://www.goodreports.org/reporting-checklists/consort/info/#24) | Where the full trial protocol can be accessed, if available | 5 |
| Funding | [#25](https://www.goodreports.org/reporting-checklists/consort/info/#25) | Sources of funding and other support (such as supply of drugs), role of funders | 12 |

# Appendix 2. CHEERS checklist.

This appendix uses a checklist published as part of the CHEERS guidelines.^2^

|  |  | Reporting Item | Page Number |
| --- | --- | --- | --- |
| Title |  |  |  |
|  | [#1](https://www.goodreports.org/reporting-checklists/cheers/info/#1) | Identify the study as an economic evaluation or use more specific terms such as “cost-effectiveness analysis”, and describe the interventions compared. | 1 |
| Abstract |  |  |  |
|  | [#2](https://www.goodreports.org/reporting-checklists/cheers/info/#2) | Provide a structured summary of objectives, perspective, setting, methods (including study design and inputs), results (including base case and uncertainty analyses), and conclusions | 3 |
| Introduction |  |  |  |
| Background and objectives | [#3](https://www.goodreports.org/reporting-checklists/cheers/info/#3) | Provide an explicit statement of the broader context for the study. Present the study question and its relevance for health policy or practice decisions | 4 |
| Methods |  |  |  |
| Target population and subgroups | [#4](https://www.goodreports.org/reporting-checklists/cheers/info/#4) | Describe characteristics of the base case population and subgroups analysed, including why they were chosen. | 5,6,7 |
| Setting and location | [#5](https://www.goodreports.org/reporting-checklists/cheers/info/#5) | State relevant aspects of the system(s) in which the decision(s) need(s) to be made. | 5,7 |
| Study perspective | [#6](https://www.goodreports.org/reporting-checklists/cheers/info/#6) | Describe the perspective of the study and relate this to the costs being evaluated. | 4,7 |
| Comparators | [#7](https://www.goodreports.org/reporting-checklists/cheers/info/#7) | Describe the interventions or strategies being compared and state why they were chosen. | 5,6 |
| Time horizon | [#8](https://www.goodreports.org/reporting-checklists/cheers/info/#8) | State the time horizon(s) over which costs and consequences are being evaluated and say why appropriate. | 7 |
| Discount rate | [#9](https://www.goodreports.org/reporting-checklists/cheers/info/#9) | Report the choice of discount rate(s) used for costs and outcomes and say why appropriate | 7 |
| Choice of health outcomes | [#10](https://www.goodreports.org/reporting-checklists/cheers/info/#10) | Describe what outcomes were used as the measure(s) of benefit in the evaluation and their relevance for the type of analysis performed | 6,7 |
| Meaurement of effectiveness | [#11a](https://www.goodreports.org/reporting-checklists/cheers/info/#11a) | Single study-based estimates: Describe fully the design features of the single effectiveness study and why the single study was a sufficient source of clinical effectiveness data | 5-8 |
| Measurement of effectiveness | [#11b](https://www.goodreports.org/reporting-checklists/cheers/info/#11b) | Synthesis-based estimates: Describe fully the methods used for identification of included studies and synthesis of clinical effectiveness data | NA |
| Measurement and valuation of preference based outcomes | [#12](https://www.goodreports.org/reporting-checklists/cheers/info/#12) | If applicable, describe the population and methods used to elicit preferences for outcomes. | 7 |
| Estimating resources and costs ** | [#13a](https://www.goodreports.org/reporting-checklists/cheers/info/#13a) | Single study-based economic evaluation: Describe approaches used to estimate resource use associated with the alternative interventions. Describe primary or secondary research methods for valuing each resource item in terms of its unit cost. Describe any adjustments made to approximate to opportunity costs | 7 |
|  |  |  |  |
|  |  |  |  |
| Estimating resources and costs | [#13b](https://www.goodreports.org/reporting-checklists/cheers/info/#13b) | Model-based economic evaluation: Describe approaches and data sources used to estimate resource use associated with model health states. Describe primary or secondary research methods for valuing each resource item in terms of its unit cost. Describe any adjustments made to approximate to opportunity costs. | NA |
| Currency, price date, and conversion | [#14](https://www.goodreports.org/reporting-checklists/cheers/info/#14) | Report the dates of the estimated resource quantities and unit costs. Describe methods for adjusting estimated unit costs to the year of reported costs if necessary. Describe methods for converting costs into a common currency base and the exchange rate. | 7 |
| Choice of model | [#15](https://www.goodreports.org/reporting-checklists/cheers/info/#15) | Describe and give reasons for the specific type of decision analytical model used. Providing a figure to show model structure is strongly recommended. | NA |
| Assumptions | [#16](https://www.goodreports.org/reporting-checklists/cheers/info/#16) | Describe all structural or other assumptions underpinning the decision-analytical model. | NA |
| Analytical methods | [#17](https://www.goodreports.org/reporting-checklists/cheers/info/#17) | Describe all analytical methods supporting the evaluation. This could include methods for dealing with skewed, missing, or censored data; extrapolation methods; methods for pooling data; approaches to validate or make adjustments (such as half cycle corrections) to a model; and methods for handling population heterogeneity and uncertainty. | 7,8 |
| Results |  |  |  |
| Study parameters | [#18](https://www.goodreports.org/reporting-checklists/cheers/info/#18) | Report the values, ranges, references, and, if used, probability distributions for all parameters. Report reasons or sources for distributions used to represent uncertainty where appropriate. Providing a table to show the input values is strongly recommended. | 8,9 |
| Incremental costs and outcomes | [#19](https://www.goodreports.org/reporting-checklists/cheers/info/#19) | For each intervention, report mean values for the main categories of estimated costs and outcomes of interest, as well as mean differences between the comparator groups. If applicable, report incremental cost-effectiveness ratios. | 8,9 |
| Characterising uncertainty | [#20a](https://www.goodreports.org/reporting-checklists/cheers/info/#20a) | Single study-based economic evaluation: Describe the effects of sampling uncertainty for the estimated incremental cost and incremental effectiveness parameters, together with the impact of methodological assumptions (such as discount rate, study perspective). | 8,9 |
| Characterising uncertainty | [#20b](https://www.goodreports.org/reporting-checklists/cheers/info/#20b) | Model-based economic evaluation: Describe the effects on the results of uncertainty for all input parameters, and uncertainty related to the structure of the model and assumptions. | NA |
| Characterising heterogeneity | [#21](https://www.goodreports.org/reporting-checklists/cheers/info/#21) | If applicable, report differences in costs, outcomes, or cost effectiveness that can be explained by variations between subgroups of patients with different baseline characteristics or other observed variability in effects that are not reducible by more information. | 9 |
| Discussion |  |  |  |
| Study findings, limitations, generalisability, and current knowledge | [#22](https://www.goodreports.org/reporting-checklists/cheers/info/#22) | Summarise key study findings and describe how they support the conclusions reached. Discuss limitations and the generalisability of the findings and how the findings fit with current knowledge. | 10,11 |
| Other |  |  |  |
| Source of funding | [#23](https://www.goodreports.org/reporting-checklists/cheers/info/#23) | Describe how the study was funded and the role of the funder in the identification, design, conduct, and reporting of the analysis. Describe other non-monetary sources of support | 12 |
| Conflict of interest | [#24](https://www.goodreports.org/reporting-checklists/cheers/info/#24) | Describe any potential for conflict of interest of study contributors in accordance with journal policy. In the absence of a journal policy, we recommend authors comply with International Committee of Medical Journal Editors recommendations | 12 |

# Appendix 3. Protocol for primary endpoint outcome data collection.

Day 0 was the day of randomisation. Each participant reached the primary endpoint 364 days post-randomisation. Automated emails were sent one minute after midnight on the indicated day. Manual SMS were sent at any time on the indicated day. Telephone calls were made at any time on the indicated day.

| Day | Mechanism | Message |
| --- | --- | --- |
| 364 | Automated email | Subject: NEON – £20 for final questionnaires completion!  It’s now been at least 52 weeks since you signed up for NEON, and we have a final set of questionnaires for you to fill out. The information that you provide lets us work out how effective NEON has been at helping people. You can claim a £20 voucher once you have completed all questionnaires.  It’s really important for our trial that you fill these out even if you haven’t used NEON much or at all. Log in at <https://recoverystories.uk/login>. You can reset your password at <https://recoverystories.uk/reset> if you need to. Email [neon@nottingham.ac.uk](mailto:neon@nottingham.ac.uk) for support, or text 07973 841271.  If you’re in the control group, you’ll get access to all of the recovery stories once you’ve completed the questionnaires. |
| 371 | Automated email | Subject: NEON – Reminder: £20 for completing due questionnaires!  This email is just to remind you that we have a final set of questionnaires for you to fill out. This data is essential for the success of our trial. You can claim a £20 voucher once you have completed all questionnaires.  Log in at <https://recoverystories.uk/login>. You can reset your password at <https://recoverystories.uk/reset> if you need to. Email [neon@nottingham.ac.uk](mailto:neon@nottingham.ac.uk) for support. |
| 375 | Manual SMS | Be part of health research! Your final NEON questionnaires are due which means £20 payment if completed. Log in: recoverystories.uk/login. Need help? Email [neon@nottingham.ac.uk](mailto:neon@nottingham.ac.uk) /text 07973 841271 |
| 378 | Automated email | Subject: NEON – how we use your questionnaires  Some final questionnaires for NEON are waiting for you. We use the data from these questionnaires to compare the experiences of people who have been given immediate access to recovery stories and people who get delayed access. You can claim a £20 voucher once you have completed all questionnaires.  It’s really important for our trial that you fill these out even if you haven’t used NEON much or at all. Log in at <https://recoverystories.uk/login>. You can reset your password at <https://recoverystories.uk/reset> if you need to. Email [neon@nottingham.ac.uk](mailto:neon@nottingham.ac.uk) for support, or text 07973 841271. |
| 385 | Automated email | Subject: NEON – £20 voucher for 14 questions  You can now receive a £20 voucher for completing only our first questionnaire, which has 14 simple questions.  Log in at <https://recoverystories.uk/login>. |
| 388 | Text message  Researcher follow-up 1 | **20 minutes prior to telephone call send the following text:**  Hi, I'm [name], a researcher at the University of Nottingham from the NEON study that you might remember signing up to a while ago.  This is just to let you know I'll be calling you from this number this afternoon about your final questionnaire.  We're sending a £20 voucher to everyone who completes the questionnaire - it takes less than 5 minutes. It's a really important one for our study so we'd really appreciate it! :) You can do it on the phone with me or via email, text or logging in at recoverystories.uk  THANK YOU, your contribution to our health research is so important ☺  And please do let me know if there is a better time to call you - all the best, [name] |
| 388 | Telephone call  Researcher follow-up 1 | Hi there, My name is [name], I’m a researcher at the University of Nottingham, on the NEON study. I texted you earlier saying that I was going to call you about the final NEON questionnaires, you might have seen it? Have you got a couple of minutes for a quick chat please?  *If not a good time -* Is there a better time to call back? Or you could also complete the questionnaire online/by text/email? >> *Log preferences on the spreadsheet & take any actions e.g. send log-in reminder, send MANSA by text or email*  *If a good time -* This number was given as a contact by someone who’s been involved in our study, NEON. You would have signed up to the study about a year ago - it was about accessing other people’s stories online, can I check if that was you? [nb don’t mention mental health at this stage]  [**Option 1: Person doesn’t recall NEON**]  Ok thank you for your time. We’ll make sure not to call this number again.  [**Option 2: Person confirms we are speaking to the correct person**]  Thanks, it’s about your final questionnaire for the trial. It’s quite an important one for the study, so we’re calling people to let them know there are different options for filling it in. So we could do it together on the phone now – there are 12 questions – or I could email or text it to you, or you could do it online. Would you be happy with any of those options?  **[Option 1: If happy with completion by phone call]**  That’s great, thank you. *Start the questionnaire*  *Questionnaire end –* That’s all the questions I have for you. Do you have any for me? *[answer questions if any]*. I will pass on your email address to my colleagues to process the voucher payment. Can you just confirm your email address for me please? You will receive the voucher via email in about 1-2 weeks time. And just to ask you not to request a second voucher if you do log on again and complete the questionnaires online.  **[Option 2: if text/email/online completion preferred**]  Thank you, we’re sending everyone a £20 voucher as a small thank you – once we receive your responses we’ll get that sent to you, it should take around 1-2 weeks to arrive. Can you just confirm your email address for me please? >> *End call, then send MANSA via email/text or email the login as a reminder to complete online]*  **What to do if you get through to voicemail**  Don’t leave a message. Instead, text & call again 2 days later.  **What to do if the person is angry about the texts/calls at any stage**  Let them know we won’t call again |
| 390 | Automated email | Subject: NEON – two days left to complete questionnaires [to earn £20]  There’s only two days left to receive a £20 voucher for completing only our first questionnaire, which has 14 simple questions.  Log in at <https://recoverystories.uk/login>.  You can email [neon@nottingham.ac.uk](mailto:neon@nottingham.ac.uk) if you’d like us to call you for your responses instead, or text 07973 841271  Or you can complete the questionnaire by responding to this email.  To complete it, you just need to leave in the answer in the questions below that matches your response to each question, and delete the other responses.  So for example, for question 1, if you’re employed, you would delete all text except “*1 = employed*”.  Please then send your responses back to this email address.  We’ll then send you a £20 voucher as a small thank you for your time (it will take around 1-2 weeks to arrive).  Just to let you know (as some of the questions are quite personal), that the data will be anonymised when we enter it into our trial database.  **Pre-question 1: What is your occupation?**  *1=Employed 2=Sheltered employment 3=Training and education 4=Unemployed 5=Retired*  **Pre-question 2: Do you live alone or with others?**  *1=Alone 2=With Others*  **Q1: How satisfied are you with your life as a whole today?**  *1=Couldn’t be worse 2=Displeased 3=Mostly dissatisfied 4=Mixed 5=Mostly satisfied 6=Pleased 7=Couldn’t be better*  **Q2: How satisfied are you with your job / sheltered employment / training / education as your main occupation?  Or if unemployed or retired - how satisfied are you with being unemployed / retired?**  *1=Couldn’t be worse 2=Displeased 3=Mostly dissatisfied 4=Mixed 5=Mostly satisfied 6=Pleased 7=Couldn’t be better*  **Q3: How satisfied are you with your financial situation?**  *1=Couldn’t be worse 2=Displeased 3=Mostly dissatisfied 4=Mixed 5=Mostly satisfied 6=Pleased 7=Couldn’t be better*  **Q4: How satisfied are you with the number and quality of your friendships?**  *1=Couldn’t be worse 2=Displeased 3=Mostly dissatisfied 4=Mixed 5=Mostly satisfied 6=Pleased 7=Couldn’t be better*  **Q5: How satisfied are you with your leisure activities?**  1=Couldn’t be worse 2=Displeased 3=Mostly dissatisfied 4=Mixed 5=Mostly satisfied 6=Pleased 7=Couldn’t be better  **Q6: How satisfied are you with your accommodation?**  *1=Couldn’t be worse 2=Displeased 3=Mostly dissatisfied 4=Mixed 5=Mostly satisfied 6=Pleased 7=Couldn’t be better*  **Q7: How satisfied are you with your personal safety?**  *1=Couldn’t be worse 2=Displeased 3=Mostly dissatisfied 4=Mixed 5=Mostly satisfied 6=Pleased 7=Couldn’t be better*  **Q8: How satisfied are you with the people that you live with? If you live alone, how satisfied are you with living alone?**  *1=Couldn’t be worse 2=Displeased 3=Mostly dissatisfied 4=Mixed 5=Mostly satisfied 6=Pleased 7=Couldn’t be better*  **Q9: How satisfied are you with your sex life?**  *1=Couldn’t be worse 2=Displeased 3=Mostly dissatisfied 4=Mixed 5=Mostly satisfied 6=Pleased 7=Couldn’t be better*  **Q10: How satisfied are you with your relationship with your family?**  *1=Couldn’t be worse 2=Displeased 3=Mostly dissatisfied 4=Mixed 5=Mostly satisfied 6=Pleased 7=Couldn’t be better*  **Q11: How satisfied are you with your physical health?**  *1=Couldn’t be worse 2=Displeased 3=Mostly dissatisfied 4=Mixed 5=Mostly satisfied 6=Pleased 7=Couldn’t be better*  **Q12: How satisfied are you with your mental health?**  *1=Couldn’t be worse 2=Displeased 3=Mostly dissatisfied 4=Mixed 5=Mostly satisfied 6=Pleased 7=Couldn’t be better* |
| 395 | Text message  Researcher follow-up 2 | Use same text as day 388 |
| 395 | Telephone call  Researcher follow-up 2 | Use same script as day 388. |
| 402 | Text message  Researcher follow-up 3 | Use same text as day 388 |
| 402 | Text message  Researcher follow-up 3 | Use same script as day 388. |
| 409 | Text message | Use same text as day 388 with addition of text in red  Hi, I'm [name], a researcher at the University of Nottingham from the NEON study that you might remember signing up to a while ago.  This is just to let you know I'll be calling you from this number this afternoon about your final questionnaire. We won’t call again after today.  We're sending a £20 voucher to everyone who completes the questionnaire - it takes less than 5 minutes. It's a really important one for our study so we'd really appreciate it! :) You can do it on the phone with me or via email, text or logging in at recoverystories.uk  THANK YOU, your contribution to our health research is so important ☺  And please do let me know if there is a better time to call you - all the best, [name] |
| 409 | Telephone call | Use the same script as day 388. |

# Appendix 4. Description of the NEON Collection and the NEON Intervention

## The NEON Collection

The NEON Collection is diverse collection of mental health recovery narratives. Each narrative is presented in a single digital file, such as a PDF, am image, or a video. To create the NEON Collection, we identified appropriate curation procedures,^3-6^ including principles for establishing informed consent. We used these curation procedures to assemble a diverse collection of recovery narratives,^7^ including chapters from narrative collections published as books,^8, 9^, online videos,^10^ text-based narratives,^11^ and interview recordings.

Access to the NEON Collection requires a username and password, so that narrative content cannot be indexed by automated services such as Google <https://www.google.com/>. This was a deliberate choice, to support the comfort of NEON Collection narratives.

We have included narratives from 32 published collections. These include the web-based collections OC87 <https://oc87recoverydiaries.org/> and The Schizophrenia Oral History Project <https://schizophreniaoralhistories.com/>, and selected narratives from published books including Psychosis: Stories of Recovery and Hope,^12^ Riding the Storms,^9^ The Colour of Madness,^8^ and a series of books conceptualising recovery as spiritual emergence published by the organisation Emerging Proud <https://emergingproud.com/>. Most narratives were in the public domain already, but some had not been previously published and were donated directly to the study specifically for use in the NEON Trial. A small number of included narratives were donated by NEON trials participants who described feeling empowered to author a narrative.

Every narrative was assessed for inclusion, and was characterised using the Inventory of the Characteristics of Recovery Stories (INCRESE),^13^ by a researcher who examined the narrative in full. The characterisation process allowed us to identify potentially distressing content and hence to assign content warning to relevant narratives.^6, 14^ A distinguishing feature of the NEON Collection is that content warnings support self-management of engagement, for example through enabling users to avoid forms of content that they might find distressing for idiographic reasons.

The NEON Trial opened with 348 narratives, and (per protocol) narratives were added during the trial period, with 659 narratives available when the final participant reached the primary endpoint. We chose not to limit narratives to those including content about psychosis, informed through deliberations with LEAP.

Inclusion and exclusion criteria for narratives in the NEON Collection are as follows (summarised from <https://www.researchintorecovery.com/neoncollection/>, accessed 1 April 2022).

### Inclusion criteria

(1) includes elements of adversity or struggle that relate to mental health problems, broadly defined

(2) includes descriptions of strengths, successes or survival, as defined by the narrator, or identifiable by a third-party

(3) refers to events or actions over a period of time. “Events” can include external events or internal states (e.g. changes in mood, perception, perspective)

(4) is told by a single individual with experience of mental health problems and self-defined recovery

(5) where speech or words are used, is mainly in English, or if translated the translation needs to be provided or approved by the narrator

(6) Either (a) is provided in a digital file OR (b) is provided in a format which can easily be converted into a digital file OR (c) is hosted on an existing web-page, the URL to the web-page is permanent, and the page does not contain links that would enable navigation to another page.

### Exclusion criteria

(1) is presented as fictional

(2) is told by anyone other than the individual experiencing mental health problems and recovery, e.g. if told entirely by a carer or journalist

(3) (for video and audio narratives) the quality of recording is so low that the narrative is very difficult or not possible to understand

(4) uses a multimedia approach which cannot easily be integrated into a single file or format

(5) contains descriptions of potentially harmful behaviours in sufficient detail as to be likely to encourage imitation

(6) indicates that the narrator has engaged in a previously undisclosed serious criminal activity

(7) the narrator is a child or appears to be a child, unless it has been confirmed that the narrator is now an adult and has provided consent for a childhood story to be shared

(8) contains hate speech

(9) provides information about a third party which might reasonably lead to harm being caused to the third party such as providing directly identifying information about someone accused of abuse

(10) includes sensitive personal information about individual third parties, unless the third party has already made this information public, e.g. by publishing their own recovery story, or unless the third party is no longer alive. A narrative includes sensitive information (as defined in UK data protection law) about a third party if it clearly reveals their political or religious beliefs, mental or physical health conditions, sexual orientation or behaviours, or any offences committed or alleged to have been committed by them

(11) reveals the adoption status of a third party, unless the third party has already made this information public

(12) raises any other unforeseen concerns

## NEON Intervention

The NEON Intervention is a web-application providing access to the NEON Collection of recovery narratives. The development of the NEON Intervention was informed by theory on recovery narrative characteristics ^13, 15-17^ and impact pathways.^6, 18-20^ The NEON Intervention is accessed through a web-browser on a smartphone or computer. It was engineered to work on the broadest range of technologies possible. This included communal computers provided in public settings such as libraries, due to concerns around digital exclusion.^21^ For the NEON Trial, access to the NEON Intervention was provided immediately after randomisation for those allocated to the intervention arm, or after the primary endpoint for those allocated to the control arm.

Before their first narrative access through the NEON Intervention, participants were presented with orienting information that describes the purpose of the NEON Intervention, and asked to complete an updatable personal profile defining narrative formats and content they wished to avoid (e.g., text, self-harm, violence). Initial contact with a mental healthcare technology can be challenging,^22^ and so to orient them to the system participants were shown a first narrative identified empirically as being hope-promoting for participants in a prior study^23^ and not requiring content warnings. Selection of the first narrative respected participant format preferences e.g. a video narrative was shown to participants wishing to avoid text.

After receiving the first narrative, and all subsequent narratives, participants rated immediate impact by responding to one mandatory and four optional validated narrative feedback questions.^23^ The mandatory question asked “How hopeful did the story leave you feeling?”. Available responses were: Less hopeful than before, No change, A bit more hopeful, Much more hopeful. After responding, participants received access to the NEON Intervention homepage, with four narrative access mechanisms: (1) Match me to a story; (2) Get me a random story; (3) Browse stories; (4) My Stories. The home page is shown in figure S1.

Of the four options on the homepage, *Match me to a story* selects a narrative not previously accessed, by invoking the recommender system. *Get me a random story* selects a narrative not previously accessed, using a random number generator. *Browse stories* allows the selection of a narrative using demographic and content categories derived from INCRESE, and illustrated in figure S2 and figure S3. *My stories* allowed return access to narratives previously rated as hope-inspiring or bookmarked by the participant.

If a user requests a narrative through any of these options, and if that narrative contains a content warning, then a screen describing the content warning is first presented, enabling the user to choose whether to progress to the narrative or not. If they choose to progress to the narrative, then it is displayed on a single page. At the bottom of the page is a small interface encouraging the user to provide narrative feedback.

The homepage also included links to a page providing signposting and self-help information for participants experiencing distress, and a separate trial information page. Control arm participants received access to a homepage that retained distress and trial information, but which excluded narrative access mechanisms until primary endpoint questionnaires were completed at 52-week follow up. All NEON Intervention usage by trial participants was logged, including use of narrative access routes, responses to narrative feedback questions, and access to study information.


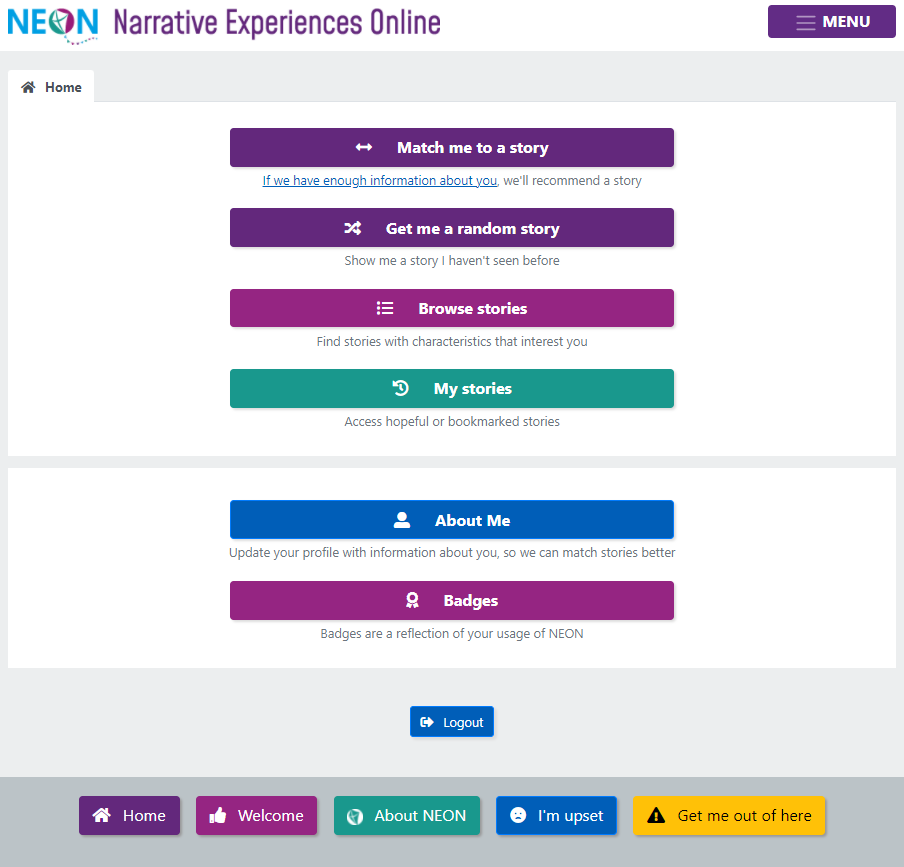


Figure S1. NEON Intervention home page


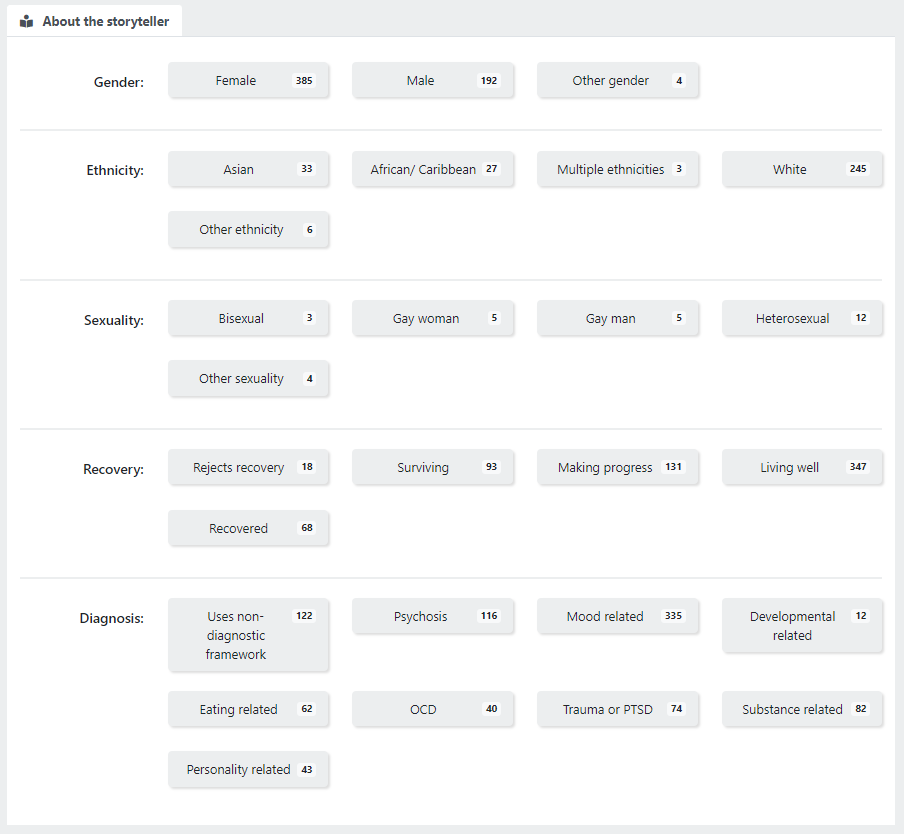


Figure S2. Categories relating to the storyteller displayed on the Browse Stories option


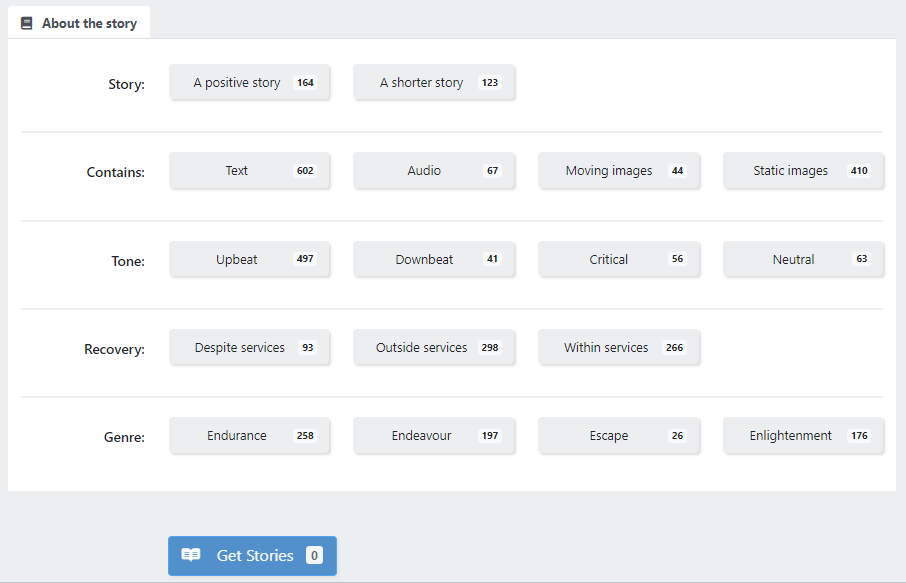


Figure S3. Categories relating to the story displayed on the Browse Stories option

The hybrid recommender system utilised: (A) personal profiles; (B) INCRESE characteristics; and (C) narrative feedback ratings. It was initially trained with feasibility study usage data.^23^ INCRESE characteristics were used to identify similar narratives to those rated positively by the participant (content-based recommendation) using a k-Nearest Neighbour (kNN) filtering algorithm, with a view to tackle the cold-start problem in scenarios where available ratings by users and for narratives were low. Participant profiles were used to identify similar participant profiles, and then to identify narratives rated positively by these participants (collaborative recommendation) using Singular Value Decomposition (SVD) and SVD++ filtering algorithms, where an adequate number of ratings was available. The narrative with the highest estimated rating was selected from a combined list. The recommender system was found to deliver a better-than-random performance in our performance evaluation.^24^

From the start of the trial, messages to enable engagement were emailed to intervention arm participants, and sent by Short Messaging Service (SMS) to participants who had provided a mobile number. All messages are in appendix 5. Our over-arching expectation was that participants should self-manage their engagement, for example by practicing self-compassion (e.g. understanding and kindness towards oneself) if they found narratives distressing, and hence engagement messages avoided language that directed use. During the trial, we added further functionality to enable engagement. This consisted of anonymous participant testimonials, badges (graphical symbols received on meeting thresholds such as 10 narrative requests), and a system for capturing personal reflections on impactful narratives. We used several harm reduction strategies.^23^ Narratives containing potentially distressing content could be hidden, content warnings were displayed if relevant on remaining narratives, individual narratives could be hidden, and participants could identify and be reminded of personal self-management strategies. All pages included buttons to access guidance for those experiencing distress, and to immediately leave the NEON Intervention.

# Appendix 5. NEON Intervention engagement messages.

| ID | Form | Date | Message |
| --- | --- | --- | --- |
| 1 | Email | 22/07/20 | Subject: New Stories Added to NEON  Dear NEON trial participant,  We hope you’re doing ok today. We wanted to let you know that on Monday 22nd June we added 68 new stories to NEON, and there are now 424 stories of recovery on the website.  The log in page is here: recoverystories.uk  If you’ve forgotten your password, you can reset it here: recoverystories.uk/reset  We hope you’re finding some of the stories helpful. The NEON team is continuing to search for new stories, so it might be worth checking back in if you haven’t logged on for a while. And, of course, you can use NEON as little or as much as you want until the end of the trial. It’s up to you!  If there are stories from particular perspectives that you would like to see more of, please contact the team at neon@nottingham.ac.uk.  Thank you for your continuing participation in the NEON trials. You are making a direct contribution to health research at a very difficult time and we really appreciate it.  With our best wishes from the NEON team. |
| 2 | Email | 09/09/20 | Subject: New NEON stories + Donate Your Story  Dear NEON trial participant,  We hope you’re doing ok today. We wanted to let you know that on Wednesday 26th August we added 64 new stories to NEON, and there are now 488 stories of recovery on the website. We’ll continue to add new stories to give you the best chance of finding a story that might help you.  The log in page is here: recoverystories.uk  If you’ve forgotten your password, you can reset it here: recoverystories.uk/reset  We hope you’re finding some of the stories helpful. The NEON team is continuing to search for new stories, so it might be worth checking back in if you haven’t logged on for a while. And of course, you can use NEON as little or as much as you want until the end of the trial. It’s up to you!  If you would like to offer your own story for use in our trials, then you can do this at <http://researchintorecovery.com/donateastory>.  Thank you for your continuing participation in the NEON trial. You are making a direct contribution to health research at a very difficult time and we really appreciate it.  With our best wishes from the NEON team. |
| 3 | Email | 08/10/20 | Subject: NEON Trials – 6-month progress (with attached pdf)  Dear NEON trial participant,  We hope you are doing ok today. We’re emailing to thank you for your ongoing participation in our study. We’ve been recruiting for 6 months and have seen a huge increase in participation. Please find attached a summary of our progress so far.  We’re still looking for trial participants so if you know anyone who is aged 18 and over, lives in England and   - Has experience of psychosis or similar (e.g. hearing voices, hallucinations, non-consensus realities) - Has experience of other mental health problems   or   - Cares for someone with mental ill health experiences   Please help us spread the word by telling them about https://recoverystories.uk  There are currently over 480 stories in NEON, and we’re constantly looking to add more stories, giving you the best chance of finding a story that might help. If you haven’t logged in for a while, you could check back to see if there are any new stories for you.  To log in, please head to: https://recoverystories.uk  You can reset your password at: https://recoverystories.uk/reset  Remember, you can use the NEON Intervention as little or as much as you’d like, it is completely up to you.  Do you, or someone you know, have a story to offer to the NEON trials? You can find out more on our Donate a story page, where you’ll also find a guide for people interested in sharing their mental health experiences published by our colleagues at the McPin Foundation.  We believe that NEON will be most effective if our stories are diverse. All stories are welcome, and we are particularly looking for stories in the following categories:   - Stories where the teller’s ethnicity is anything other than White British - Stories where the teller identifies as being neurodiverse, with a focus on people who identify with ADHD, Autism, dyslexia, dyspraxia or similar - Stories where the teller talks about their sexuality - Stories where the teller identifies as trans, intersex or non-binary   Which might be less heard than others.  Thank you for your continuing participation in the NEON trials. You are making a direct contribution to research as a very difficult time and we really appreciate it.  If you have any questions, please do not hesitate to get in touch.  With our best wishes, |
| 4 | Email | 16/12/20 | Subject: NEON is still available  Hi, and thank you for taking part in our trials.  The NEON Intervention will be up and running until end of September 2022, and you can log in at any point to look at our stories using <https://recoverystories.uk/login>.  Following some feedback from someone in our trials, we’ve updated the “Browse stories” function to make it easier to find stories where people describe themselves as “Recovered”, and we’ll be adding some new stories in the next few days.  We hope that you can find some value for yourself in the work of our story donors and our team.  Best wishes, the NEON team.. |
| 5 | Email | 28/01/21 | Subject: NEON Trials - Your Registration  Hi!  Thank you for registering for the NEON trials. Every time you log into NEON we learn more about how recovery stories can help people. Here’s some news and reminders of how to access the NEON website if you need it.  You can log into NEON at [recoverystories.uk/login](https://recoverystories.uk/login) or reset your password at [recoverystories.uk/reset](https://recoverystories.uk/reset). If you are having difficulties accessing NEON you can email us at [neon@nottingham.ac.uk](mailto:neon@nottingham.ac.uk).  We thought you’d like to know that the NEON trials were recently on both BBC Radio 4 and BBC South East regional news, featuring people talking about their experiences of using the NEON website. You can listen again at <https://www.bbc.co.uk/programmes/m000pfh3> and <https://bit.ly/2MlQxoC>  We regularly add new stories to the NEON website. There are now over 500 stories, with more to come. These stories cover a really broad range of experiences. All stories describe both difficulties and successes that people have had. Topics covered by the stories include   - strategies and tips that have worked - just getting through, and what helps people survive in the hardest times - a change of meaning or perspective in people’s lives - leaving behind approaches that just haven’t worked   There are stories of recovery both within and outside of NHS services; stories about hope, and others that are more about letting us know that we are not alone. There are text, video, audio and image-based stories, long and short, from people around the world.  We hope you’ll find someone’s story that speaks to your own situation today.  Apologies if you received a poorly-formatted version of this email yesterday, this was a technical problem which we have now fixed.  All the best, The NEON Team |
| 6 | Email | 28/01/21 | Have you signed up to a NEON trial and been given immediate access to our collection of recovery stories? Remember that you can log in at [https://recoverystories.uk/login](https://recoverystories.uk/login%20) to access stories.  Your password can be reset at [https://recoverystories.uk/reset](https://recoverystories.uk/reset%20) or contact neon@nottingham.ac.uk if experiencing technical problems. |
| 7 | Email | 11/02/21 | Subject: New stories added to NEON  NEON now has almost 600 stories, and whilst we know that they can’t make a difference to everyone, you might find something in there that helps. Login at <https://recoverystories.uk/>login, reset your password at <https://recoverystories.uk/reset>, or contact neon@nottingham.ac.uk if you need help with technical problems.  Lara Tozer, who talks about NEON at https://bit.ly/2MlQxoC has said that “it gives me inspiration reading, watching, listening… stories from other people who have recovered or are recovering”, and the NEON study team is very proud that we’ve made even just a small amount of difference in the world. |
| 8 | Email | 18/02/21 | Hi from NEON. We have added 100 stories, have a look at https://recoverystories.uk/login. If you have any technical problems text us back. |
| 9 | Email | 24/02/21 | Subject: Feedback on NEON  Dear NEON Intervention Users,  Just a quick reminder that your place in the NEON intervention is still ongoing. You can log in at <https://recoverystories.uk>. If you need to reset your password, please click [here](https://recoverystories.uk/reset).  As a valued user of the NEON intervention, we would like some feedback from you on how we can improve the intervention (for example; are there any barriers that make you less likely to use the intervention?). If you have a few minutes, please send us an email to let us know your thoughts to neon@nottingham.ac.uk! |
| 10 | Email | 11/03/21 | Subject: NEON – some thoughts from a participants  Hi NEON Intervention Users,  Have you ever wondered how other people are finding the NEON Intervention?  Listen to our mini-interviews with Lara, who uses the NEON intervention. We talk about when she uses the NEON intervention <https://vimeo.com/518074107>, how she finds a story <https://vimeo.com/518073066>, and the benefits she has experienced from using NEON <https://vimeo.com/518073389>.  ‘It makes you feel like you are not alone, like you are not the only one experiencing these issues’ – Lara  Be part of the NEON Intervention community today, by signing in and exploring the range of stories available. Finding a story is quick, easy and simple!  All the best!  NEON Team  You can log in at <https://recoverystories.uk>. If you need to reset your password: [https://recoverystories.uk/reset](https://recoverystories.uk/reset%20) . |
| 11 | Email | 25/03/21 | Dear NEON intervention users,  Hope you’re doing ok this week.  We’ve been asking for feedback recently – **big thanks** to you if you emailed or spoke to us!  One question people had was how to find stories of a particular kind. There’s a ‘**browse stories’** option on the home page which lets you search for stories by category.  You can find a video demonstrating how to use this [here](https://vimeo.com/527296181). We show 4 examples of how to search for different combinations: (1) depression/anxiety stories from Asian women (2) men’s PTSD stories (3) text-based transformation stories from outside mental health services (4) all video-based stories.  But what do the categories mean?  You can search by narrator’s gender, ethnicity, sexuality, type of mental health issue experienced & where they’re at in terms of recovery. You can also search by story type:  **format** - text (article, poem, chapter), audio (podcast), moving image (video or film) or static image (picture)  **tone** – sometimes we’re in the mood for something upbeat.  Other times it can help to have more neutral, critical, or even downbeat stories – e.g. it can help us feel less alone to read/hear about someone else’s difficult times  **recovery** – some narrators find mental health services useful in their recovery, some find other things/services more useful, some described recovering despite accessing mental health services that didn’t help.  **genre** – ‘Endurance’ stories describe struggle and challenge.  ‘Endeavour’ stories contain strategies & tips that have worked for people. ‘Escape’ stories describe surviving damaging services, or other damaging contexts.  ‘Enlightenment’ stories describe transformations or changes of perspective of various kinds.  We hope these categories help you to find a story that supports you today.  You can log in at <https://recoverystories.uk>.  If you need to reset your password, please click [here](https://recoverystories.uk/reset).  We send occasional reminders about NEON by SMS. If you'd like receive these, please add your mobile number through the "About me" page.  All the best,  The NEON team |
| 12 | SMS | 25/03/21 | Hi from NEON, we’ve added a video on how to browse through stories, look here:  [https://vimeo.com/527296181. Try it out at https://recoverystories.uk](https://vimeo.com/527296181.%20Try%20it%20out%20at%20https:/recoverystories.uk)/ |
| 13 | Email | 08/04/21 | Subject: NEON- spotlight on three stories  Dear all,  We have a new feature on the NEON intervention to tell you about today. Story Spotlight enables us to direct you to particular stories, and if you’ve not yet tried out the website it might give you some places to start.  We know that people find many different ways to recover from mental health distress. The stories in the NEON Collection reflect that. Here are three, for example, from people with very different experiences of mental health services:  Someone who found services helpful: [https://recoverystories.uk/showStory?story=223](%20https:/recoverystories.uk/showStory?story=223%20)  Someone who found services made things worse: [https://recoverystories.uk/showStory?story=535](https://recoverystories.uk/showStory?story=535%20)  Someone whose story doesn’t mention services at all: <https://recoverystories.uk/showStory?story=420>  (If you are not logged in to NEON when you click on the link, you will be prompted for your username and password before going direct to that story, and if you have any questionnaire responses due you’ll need to complete these first, and then click on the link again).  Find more by using these categories on the “Browse stories” page: ‘within services’, ‘despite services or ‘outside services’.  We hope you find a story that suits you today.  All the best – the NEON team |
| 14 | Email | 27/04/21 | Subject: New videos from NEON  Dear NEON intervention users,  We hope you are doing ok this week. We have added some videos to the NEON website if you would like to check them out at [https://recoverystories.uk.](https://recoverystories.uk/) You can watch some tutorials about how to use NEON, find out about how the NEON study has helped others and meet some of the members of the NEON team.  If you have any questions about the trial or your participation, please feel free to contact the NEON team via email ([neon@nottingham.ac.uk](mailto:neon@nottingham.ac.uk)).  With our best wishes,  The NEON team |
| 15 | SMS | 27/04/21 | Hi from NEON check out <https://recoverystories.uk> for some new videos on how to use NEON, how the study has helped others and meet the NEON team. |
| 16 | Email | 10/05/21 | Subject: NEON – Not everyone agrees with the concept of recovery  Dear all,  For this week’s story spotlight, we want to highlight the different perspectives that are in the NEON Collection. Not everyone agrees with the concept of recovery and many people define recovery in their own terms. Read these stories to understand how people define recovery differently.  <https://recoverystories.uk/showStory?story=55>  <https://recoverystories.uk/showStory?story=74>  <https://recoverystories.uk/showStory?story=221>  (If you are not logged in to NEON when you click on the link, you will be prompted for your username and password before going direct to that story, and if you have any questionnaire responses due you’ll need to complete these first, and then click on the link again).  Find more by using these categories on the “Browse stories” page: ‘within services’, ‘despite services or ‘outside services’.  We hope you find a story that suits you today.  All the best – the NEON team |
| 17 | Email | 20/05/21 | Subject: NEON  Dear NEON intervention users,  We hope that you are doing ok this week. Thank you for your ongoing participation in the NEON trials.  Would you like to check out a recovery story today?  If you want to check out some stories you can log in at <https://recoverystories.uk>. If you need to reset your password go to <https://recoverystories.uk/reset>.  If you have any questions about the trial or your participation, please feel free to contact the NEON team via email (neon@nottingham.ac.uk).  With our best wishes,  The NEON team |
| 18 | Email | 26/05/21 | Subject: It’s hard work signing up to a trial – thank you!  Dear NEON study trial participants,  We really appreciate the time and effort it takes to sign up to an NHS clinical trial. To get to the stage you’ve reached, you’ve already done a lot of work, completing forms and questionnaires and answering questions which may not have been easy to think about.  So firstly, thank you for all of that effort! It is genuinely appreciated. Secondly, we’re sending this message to everyone in the trial who hasn’t yet accessed a story, or may have seen just 1. We’re wondering if there’s anything we can do to help you to access some more stories, now all the hard work of actually joining the trial is complete?  Of course, life is pretty complex for a lot of us right now, and there’s no obligation to access a certain number of stories – it’s completely up to you. But if you do have any questions, or if we can help you with any aspect of taking part, please do get in touch with the NEON team. You can email us here: neon@nottingham.ac.uk or text us: 07973 841271.  If you want to check out some stories you can log in at <https://recoverystories.uk>. If you need to reset your password go to <https://recoverystories.uk/reset>.  All the best, and thanks again for signing up -  The NEON team |
| 19 | Email | 10/06/21 | Subject: Read a story today with NEON!  Dear NEON Intervention user,  Don’t forget the NEON Intervention is still available. There are hundreds of stories to read, watch and listen to about people’s mental health journeys.  If you want to check out some stories you can log in at <https://recoverystories.uk>. If you need to reset your password go to <https://recoverystories.uk/reset>.  If you have any questions about the trial or your participation, please feel free to contact the NEON team via email (neon@nottingham.ac.uk).  Best wishes,  The NEON team |
| 20 | SMS | 10/06/21 | Hi from NEON! You could check out a story today at <https://recoverystories.uk>. If you need any help please text us back. |
| 21 | Email | 17/06/21 | Subject: NEON trial – frequently asked questions  Dear NEON trial participants,  Hi there, hope  you’re doing ok.  Did you know that we have two Frequently Asked Questions (FAQ) pages on the NEON website?  If you have questions about being part of the trial or using the intervention, you can check to see if they are answered [here](https://www.researchintorecovery.com/research/neon/neontrials/frequently-asked-questions/%20%20or%20here:) or [here](https://recoverystories.uk/howto).  If your question isn’t answered on these pages, feel free to contact us at [neon@nottingham.ac.uk](mailto:neon@nottingham.ac.uk).  Just a reminder that if you want to check out someone’s story today, you can log in here: [https://recoverystories.uk](https://recoverystories.uk/).  And if you need to reset your password go to <https://recoverystories.uk/reset>.  Thanks! All the best.  The NEON team |
| 22 | Email | 24/06/21 | Subject: Read a story today with NEON!  Dear NEON Intervention user,  Don’t forget the NEON Intervention is still available. There are hundreds of stories to read, watch and listen to about people’s mental health journeys.  If you want to check out some stories you can log in at <https://recoverystories.uk>. If you need to reset your password go to <https://recoverystories.uk/reset>.  If you have any questions about the trial or your participation, please feel free to contact the NEON team via email (neon@nottingham.ac.uk).  Best wishes,  The NEON team |
| 23 | Email | 08/07/21 | Subject: NEON- spotlight on three stories  Hi NEON intervention users,  We hope you are doing ok this week. Would you be interested in checking out some stories? NEON has many different types of story such as graphical stories, poetry or videos. We have spotlighted 3 such stories for you if you would like to check out the following links.  A graphical story: <https://recoverystories.uk/showStory?story=46>  Some poetry: <https://recoverystories.uk/showStory?story=199>  A video: <https://recoverystories.uk/showStory?story=166>  (If you are not logged in to NEON when you click on the link, you will be prompted for your username and password before going direct to that story, and if you have any questionnaire responses due you’ll need to complete these first, and then click on the link again).  If you have any questions about the trial or your participation, please feel free to contact the NEON team via email (neon@nottingham.ac.uk).  Many thanks,  The NEON Team |
| 24 | SMS | 08/07/21 | Hi from the NEON trial! Hundreds of people’s stories of different kinds of recovery are available for you, if you’d like to have a look. Check out the collection at <https://recoverystories.uk>. If you need any help please text us back. |
| 25 | Email | 15/07/21 | Subject: NEON is still here!  Dear NEON Intervention user,  Don’t forget the NEON Intervention is still available. There are hundreds of stories to read, watch and listen to about people’s mental health journeys.  If you want to check out some stories you can log in at <https://recoverystories.uk>. If you need to reset your password go to <https://recoverystories.uk/reset>.  If you have any questions about the trial or your participation, please feel free to contact the NEON team via email (neon@nottingham.ac.uk).  Best wishes,  The NEON team |
| 26 | Email | 22/07/21 | Subject: NEON spotlight on stories  Dear NEON Intervention user,  We hope that you are well this week. We wanted to highlight a few stories from the wide ranging collections we have on the NEON intervention.  There are a variety of stories of different lengths and media and here are a few examples:  <https://recoverystories.uk/showStory?story=521>  <https://recoverystories.uk/showStory?story=238>  <https://recoverystories.uk/showStory?story=260>  (If you are not logged in to NEON when you click on the link, you will be prompted for your username and password before going direct to that story, and if you have any questionnaire responses due you’ll need to complete these first, and then click on the link again).  If you have any questions about the trial or your participation, please feel free to contact the NEON team via email (neon@nottingham.ac.uk).  Best wishes,  The NEON team |
| 27 | Email | 05/08/21 | Hi from the NEON team!  NEW FEATURE! Add your own notes  You can now add your own notes to a story when you use the bookmarking feature. You can use the notes space to write reflections on a story, remind yourself why you wanted to bookmark it, or for anything else you want to record. These notes will not be visible to the research team or anyone else except you – they’re just for your own use.  To add notes to a story:  1. Tap/click the green ‘bookmark’ button underneath the story  2. A text box will appear, with the question: ‘is there a reason that you wanted to bookmark this story?’  3. Enter any text you like.  4. Tap/click ‘update bookmark’  5. You should get an ‘Updated bookmark!’ message. The story and notes will now appear on your ‘My Stories’ page  This is a completely optional activity - we hope you find it useful if you choose to use it. As ever, if you have any questions or comments, please feel free to contact the NEON team via email (neon@nottingham.ac.uk).  All the best,  The NEON Team |
| 28 | SMS | 05/08/21 | Don’t forget to log into the NEON Intervention to read stories about people’s mental health recovery stories. <https://recoverystories.uk> |
| 29 | Email | 12/08/21 | Hi from the NEON team, hope your week’s going ok.  NEW FEATURE! Badges  We’ve added a new feature to the website so you can see how you’ve used NEON so far. Simply exploring different parts of NEON can earn you a badge, which will appear on your home page. There are different kinds of badges, such as acknowledging how many stories you’ve accessed, or thanking you for trying out a particular function.  If you don’t want to use this feature, no problem - you can hide it through the About Me page at any time. As ever, if you have any questions or comments, please feel free to contact the NEON team via email ([neon@nottingham.ac.uk](mailto:neon@nottingham.ac.uk)).  All the best,  The NEON team |
| 30 | SMS | 1908/21 | Subject: NEON new feature – add notes  New feature from NEON! You can now add your own notes to stories. Click ‘bookmark’ for any stories you want to save, and the notes function will appear. Log in to try it now at <https://www.recoverystories.uk/> |
| 31 | SMS | 02/09/21 | Subject: new feature – NEON badges  Another new feature from NEON! Get badges for exploring different parts of the website. Turn off the feature on your home page if you’d rather not use it. Log in to see badges now at [https://www.recoverystories.uk](https://www.recoverystories.uk/)/ |
| 32 | Tweet | 02/09/21 | New feature for participants in @NEONTrials! You can now add your own notes to #recoverystories. Click ‘bookmark’ for any stories you want to save, and the notes function will appear. Log in to try it now at <https://www.recoverystories.uk/> |
| 33 | Tweet | 09/02/21 | Another new feature for participants in @NEONTrials! Get badges for exploring website features or different kinds of #recoverystories. Turn off the feature on your home page if you’d rather not use it. Log in to see badges now at <https://www.recoverystories.uk/> |
| 34 | Email | 16/09/21 | Subject: NEON – Meet your story match today!  Hi from the NEON Team,  Did you know that there are over 600 stories in the NEON Collection? You can access these stories by logging into [https://www.recoverystories.uk/](https://www.recoverystories.uk/%20) Browse through the stories to find your match, or let NEON do the work for you! Hit the ‘match me to a story’ button to access a story tailored to your preferences.  Have a go today!  The NEON Team |
| 35 | Email | 30/09/21 | Subject: How we developed NEON  Our NEON trials were 3 years in the making, and we did a lot of work to make NEON as good as we could. If you’d like to read about the research we did to produce NEON, you can find our papers here <https://researchintorecovery.com/neon/findings>. All of our work is freely available to all.  Remember you can always log in at [https://www.recoverystories.uk/](https://www.recoverystories.uk/%20) to access more stories. |
| 36 | SMS | 30/09/21 | Did you know NEON can show you a story with just one button click? Log in at <https://www.recoverystories.uk/>, click “get me a random story”. |
| 37 | Email | 07/10/21 | Hi from the NEON Team,  If you want to check out some stories today you can log into NEON at recoverystories.uk/login.  Have you tried engaging with stories on your phone/tablet, rather than just your computer? You can use NEON on the go if you want to use it on your mobile phone and access the 100’s of stories we have available for you.  Reading stories can be a rewarding and an emotional experience so please remember that there is always support information visible and available using the “I’m upset” button, and it has both signposts to services and provides exercises you can do if you are distressed and don’t want to talk to others.  Reset your password at recoverystories.uk/reset. If you have any questions about the trial or your participation, please feel free to contact the NEON team via email (neon@nottingham.ac.uk).  Best wishes,  The NEON team |
| 38 | Email | 14/10/21 | Subject: Reading NEON Stories  Hello there,  We hope you’re doing well. We know how hectic life can be, and we really appreciate the time you’ve taken to be a part of the NEON trial!  We hope this email can act as a refresher that NEON is available for you to use at any time, and you can find stories tailored to your preferences.  There are over 600 stories in the NEON Collection, so we hope you’ll find the story for you! If you want to give NEON another go, you can log in here [https://www.recoverystories.uk/](https://www.recoverystories.uk/%20) Browse through the stories to find your match, or let NEON do the work for you! Hit the ‘match me to a story’ button to access a story more relevant for you.  If on the other hand you’ve been experiencing any technical issues that have prevented you from using NEON, or you want to ask us about anything else, please do let us know by sending us an email to: neon@nottingham.ac.uk, and we’ll be quick to help! 😊  Thank you once again for your continued participation in NEON, we really appreciate it.  Have a great day!  The NEON Team |
| 39 | Email | 28/10/21 | Dear NEON intervention users,  Hope you’re doing ok this week.  One question people had was how to find stories of a particular kind. There’s a ‘browse stories’ option on the home page which lets you search for stories by category.  You can find a video demonstrating how to use this here. We show 4 examples of how to search for different combinations: (1) depression/anxiety stories from Asian women (2) men’s PTSD stories (3) text-based transformation stories from outside mental health services (4) all video-based stories.  But what do the categories mean? You can search by narrator’s gender, ethnicity, sexuality, type of mental health issue experienced & where they’re at in terms of recovery. You can also search by story type:  •format - text (article, poem, chapter), audio (podcast), moving image (video or film) or static image (picture)  •tone – sometimes we’re in the mood for something upbeat. Other times it can help to have more neutral, critical, or even downbeat stories – e.g. it can help us feel less alone to read/hear about someone else’s difficult times  •recovery – some narrators find mental health services useful in their recovery, some find other things/services more useful, some described recovering despite accessing mental health services that didn’t help.  •genre – ‘Endurance’ stories describe struggle and challenge. ‘Endeavour’ stories contain strategies & tips that have worked for people. ‘Escape’ stories describe surviving damaging services, or other damaging contexts. ‘Enlightenment’ stories describe transformations or changes of perspective of various kinds.  We hope these categories help you to find a story that supports you today. You can log in at <https://recoverystories.uk>. If you need to reset your password, please click [here](https://recoverystories.uk/reset).  All the best,  The NEON team |
| 40 | SMS | 28/10/21 | Real-life stories in the NEON Collection have helped over 1800 people work on their own mental health recovery. Access a story today <recoverystories.uk> |
| 41 | Email | 04/11/21 | Subject: Would you like to watch some stories on NEON?  Dear NEON Intervention Users,  Have you checked out some of our video stories on NEON?  NEON has lots of different types of story and you can login at <https://www.recoverystories.uk/>  Today we are highlighting some video stories if you would be interested to check them out using the following links:  <https://recoverystories.uk/showStory?story=779>  <https://recoverystories.uk/showStory?story=776>  <https://recoverystories.uk/showStory?story=703>  Reset your password at <https://recoverystories.uk/reset>. If you have any questions about the trial or your participation, please feel free to contact the NEON team via email (neon@nottingham.ac.uk).  Best wishes,  The NEON team |
| 42 | Email | 15/11/21 | Subject: Reading NEON Stories  Hello there,  We know how hectic life can be, and that things can get lost in the background. This email is a quick refresher that NEON is available for you to use at any time, and you can find a story in any format you like. There are video stories that you can watch whilst on the bus, audio stories to listen to whilst you take that stroll, artistic images if you’re feeling a bit more introspective, or a good old-fashioned written story to read with a hot cup of tea.  There are over 600 stories in the NEON Collection, so we hope you’ll find the story for you! If you want to give NEON another go, you can log in here [https://www.recoverystories.uk/](https://www.recoverystories.uk/%20) Browse through the stories to find your match, or let NEON do the work for you! Hit the ‘match me to a story’ button to access a story more relevant for you. There are also some categories to narrow down the topic of the story.  If on the other hand you’ve been experiencing any technical issues that have prevented you from using NEON, or you want to ask us about anything else, please do let us know by sending us an email to: neon@nottingham.ac.uk, and we’ll be quick to help! 😊  Thank you once again for your continued participation in NEON, we really appreciate it.  Have a great day!  The NEON Team |
| 43 | Email | 25/11/21 | Subject: New stories uploaded!  Dear NEON trial participants,  Hope you’re doing ok today. We wanted to let you know that we’ve uploaded 30 new stories to the NEON intervention website. We’ll continue to add new stories until the end of the trials – and did you know that you can donate your own story, as text, video, image, or audio recording? This is completely voluntary – if you’re interested, find out more here: <https://bit.ly/3pWgxZi>  And just a reminder you can log in at <https://www.recoverystories.uk/>. Or reset your password at <https://recoverystories.uk/reset>. And if you have any questions about the trial or your participation, please feel free to contact the NEON team via email (neon@nottingham.ac.uk).  All the best,  The NEON team |
| 44 | SMS | 25/11/21 | NEON is still available at <https://recoverystories.uk>, with 600 recovery stories including video, prose, poetry, and audio. More stories coming in Jan. |
| 45 | Email | 02/12/21 | Subject: NEON- individual donations spotlight on three stories  Dear all,  Most stories in NEON are from organisations or collections, however some have been donated by individuals who wanted to share their story with us. Today we would like to share with you three stories, which have been individually donated to us:  'Mummy's gone mad!' https://recoverystories.uk/showStory?story=589  Visions of Albion: A Journey Through Darkness https://recoverystories.uk/showStory?story=688  What’s Your Story Jades Story! https://recoverystories.uk/showStory?story=690  You can log in to NEON at <https://recoverystories.uk>. Or reset your password at <https://recoverystories.uk/reset>.  If you have any questions about the trial or your participation, please feel free to contact the NEON team via email ([neon@nottingham.ac.uk](mailto:neon@nottingham.ac.uk)).  Best wishes,  The NEON team |
| 46 | Email | 09/12/21 | Subject: Time for a story?  Hello!  Life can be hectic, especially with Christmas around the corner! We just wanted to send you a reminder that NEON is available for you to use at any time. There are different stories available for your liking, video stories you can watch whilst on the move, audio stories you can listen to whilst you take that stroll, artistic images to ponder if you’re feeling a bit more introspective, and of course good old-fashioned written stories you read with a hot cup of tea.  Over 600 stories are available in the NEON Collection, so we hope you’ll find the story for you! If you want to give NEON another, go, you can log in here <https://www.recoverystories.uk/> Browse through the stories to find your match, or let NEON do the work for you! Hit the ‘match me to a story’ button to access a story more relevant for you. There are also some categories to narrow down the topic of the story.  If on the other hand you’ve been experiencing any technical issues that have prevented you from using NEON, or you want to ask us about anything else, please do let us know by sending us an email to: neon@nottingham.ac.uk, and we’ll be quick to help! 😊  Thank you once again for your continued participation in NEON, we really appreciate it.  Have a great day!  The NEON Team |
| 47 | Email | 13/01/22 | Subject: NEON: new stories for a new year?  Dear NEON trial participant,  How are you doing today? If it’s a tough one, it can help to know that you’re not alone. So here’s a friendly reminder that you still have access to the Narrative Experiences Online (NEON) intervention. NEON contains over 600 real-life stories of how people have coped with their mental health, including videos, audio, text and images. Perhaps there are stories you haven’t found yet, that might help you to be kind to yourself today?  You can log in to NEON at [https://recoverystories.uk](%20https:/recoverystories.uk). Or reset your password at <https://recoverystories.uk/reset>.  If you have any questions about the trial or your participation, please feel free to contact the NEON team via email (neon@nottingham.ac.uk).  Best wishes,  The NEON team |
| 48 | SMS | 13/01/22 | Hi, don’t forget you still have access to the NEON intervention (<recoverystories.uk>) where you can read/watch/listen to over 600 mental health recovery stories from different parts of the world.  Forgotten your login details? No worries! Just head over to the <recoverystories.uk/reset> website to reset your password! |
| 49 | Email | 20/01/22 | Subject: NEON: New Year’s Resolutions  Dear NEON Intervention Users,  We hope this email finds you well and that you’ve had a great start to the new year! Is one of your new year’s resolutions to read more? Either way, NEON has lots of different types of stories, and you can login at <https://www.recoverystories.uk/> and find the one perfect for you. NEON has over 600 real-life stories of how people have coped with their mental health, including videos, audio, text and images formats.  If you experience any technical issues that prevent you from using NEON, or if you want to ask us about anything else, please send us an email at: [neon@nottingham.ac.uk](mailto:neon@nottingham.ac.uk), and we’ll be quick to help! 😊  Thank you and we wish you a fab year ahead!  The NEON Team |
| 50 | Email | 27/01/22 | Subject: The final six months of our NEON trials  The NEON trials opened in March 2020, and we spent the first year of our trials recruiting almost 2000 people who wanted to take part. We’ll be collecting people’s responses to our questionnaires up until Spring 2022, and then working on our trial reports. Once these are published, they’ll be linked to from [https://researchintorecovery.com/neon/findings.](https://researchintorecovery.com/neon/findings.%20) It can take a while to work with a journal to publish these, but we hope they’ll be available by end of 2022.  Meanwhile, NEON will remain available at <https://recoverystories.uk> until summer 2022, so there’s still time to take a look, and to see if any of our 600+ personal accounts have something in that helps you. We’re working to add new stories, and there will be more than a 100 new stories added soon.  We hope you enjoy NEON, and if you have any technical problems, you can ask for help through neon@nottingham.ac.uk. |
| 51 | Email | 10/02/22 | Subject: NEON: New stories on their way!  Have you been eagerly awaiting new stories to engage with? Over the next week the NEON team will be adding another 100 stories onto the website. In the meantime, NEON will remain available at <https://recoverystories.uk> until summer 2022, so there’s still time to take a look, and to see if any of our 600+ personal accounts have something in that helps you.  You may receive an email or text message from us reminding you to complete a final questionnaire. If you haven’t already done so, please log in to the NEON website to complete your questionnaire. All participants will be reimbursed £20 for the completed questionnaire.  If you need any technical support, please do contact us via neon@nottingham.ac.uk |
| 52 | SMS | 10/02/22 | Subject: Reading NEON Stories  Hello there,  You might remember signing up to the NEON study, which involves online recovery stories, a while ago. We know how hectic life can be, and we really appreciate the time you’ve taken to be a part of the NEON trial! This is a reminder that there are over 600 stories in the NEON Collection, so we hope you’ll find the story for you! If you want to give NEON another, go, you can log in here <https://www.recoverystories.uk/> Have a great day!  The NEON Team |
| 53 | Email | 17/02/22 | Subject: NEON’s algorithm  Did you know that NEON includes an algorithm that attempts to recommend a story for you? The design of our algorithm has been informed by 3 years of research that took place before our trials opened, and you can read about it this research here: <https://researchintorecovery.com/neon/findings>  If you want to give NEON another, go, you can log in here <https://www.recoverystories.uk/>  The NEON Team |
| 54 | Email | 24/02/22 | Subject: Pause for a personal story?  Hello there,  You might remember signing up to the NEON research study a while ago – it gives you online access to real-life stories of recovery from mental health distress. We know how hectic life can be, and we really appreciate the time you’ve taken to be a participant in the NEON trial! This is a reminder that there are 600+ stories in the NEON Collection, of many different kinds of experiences, including ones you might not expect. So we hope you’ll find a story that is relevant to your own experience! If you want to give NEON another, go, you can log in here <https://www.recoverystories.uk/>  Thanks very much and take care –  The NEON Team |
| 55 | Email | 10/03/22 | Subject: Relax with Tea and a story  Hello there,  It’s almost the end of the week, so why don’t you relax with a cuppa and a story on NEON? NEON has over 700 real-life stories of how people have coped with mental health problems, including videos, audio, text, and images. Perhaps there are stories you haven’t found yet, that might help you to be kind to yourself today?  You can log in to NEON at <https://recoverystories.uk>. Or reset your password at <https://recoverystories.uk/reset>.  If you have any questions about the trial or your participation, please feel free to contact the NEON team via email (neon@nottingham.ac.uk).  Best wishes and have a great day! 😊  The NEON Team |
| 56 | SMS | 10/03/22 | We’re in the last few months of our trials, but you still have access to NEON (<https://recoverystories.uk>) and over 700 mental health recovery stories. |
| 57 | Email | 17/03/22 | Subject: 700 stories … and yours?  Hello there,  We’ve recently uploaded some new stories to the NEON Collection, so there are now over 700 stories in total. You can search for either videos, audio, text-based or picture-based stories – we hope you’ll find one that suits you today. And if you’d like to donate your own story to the collection, you can find out more here: [researchintorecovery.com/donateastory](file:///\\ad.nottingham.ac.uk\data\Research\DRS-RRT\NEON\6.%20Governance%20and%20approvals\Phase%203\TMF\Trial%20management%20documents\Intervention%20engagement\researchintorecovery.com\donateastory)  Just a reminder that you can log in to the NEON Collection here: <https://www.recoverystories.uk/> and reset your password here: <https://recoverystories.uk/reset>  Take care and all the best –  The NEON Team |

# Appendix 6. Supplementary information about the health economics analysis

Table S1. Unit costs used to calculate healthcare resource use from Client Services Receipt Inventory data.

| CSRI Item Collected | Service | Unit cost | Description | Source |
| --- | --- | --- | --- | --- |
| In the last [6\|12] months how many nights have you stayed in hospital because of: Mental health problems | Inpatient stay per day: mental health | £428 per day | Mental health care cluster bed day cost. | PSSRU 2021 (p.34)^25^ |
| In the last [6\|12] months how many nights have you stayed in hospital because of: any other reason (e.g. physical health problems)? | Inpatient stay per day: non-mental health | £368 per day | The most recent reported excess bed day cost (£346; 2017/18 price year) was taken from NHS Reference Costs. This is calculated as the average cost per day spent in hospital exceeding the upper trim point for each HRG. This cost was then inflated to the 2020/21 price year using the most recent NHS Cost Inflation Index – Pay and Prices. The index values reported in the 2022 PSSRU document were used as they report the actual, rather than the provisional, 2020/21 index. | Reference costs 2017/18: highlights, analysis and introduction to the data (p.5)^26^  NHS Cost Inflation Index – Pay and Prices (PSSRU 2022, p.98)^27^ |
| In the last [6\|12] months how many visits have you made to: Accident and Emergency (A&E) or Minor Injuries Unit? | A&E visit | £297 per visit | Weighted average of unit costs for A&E currency codes in the 2020/21 National Schedule of NHS Costs (excluding dental and ‘dead on arrival’ currency codes). The weight is the total number of attendances per currency code divided by the total number of attendances across all currency codes. | National Schedule of NHS Costs 2020/21 (Tab: AE)^28^ |
| General practitioner/family doctor | GP visit | £39 per visit | Cost per GP surgery consultation lasting 9.22 minutes. | PSSRU 2021 (p.111)^25^ |
| Community nurse, occupational therapist, primary care counsellor, IAPT therapist OR family therapist. | Community nurse visit | £23 per visit | Band 6 community nurse (£55 per hour). Duration of visit assumption: 25-minutes (2015 PSSRU, p.175) | PSSRU 2021 (p.108)^25^  PSSRU 2015 (p.175)^29^ |
| Psychologist | Psychologist visit | £197 per visit | Non-consultant led clinical psychologist. Service code: 656 | National Schedule of NHS Costs 2020/21 (Tab: Total Outpatient Attendance)^28^ |
| Psychiatrist | Psychiatrist visit | £275 per visit | Consultant led psychiatrist. Service code: 722 | National Schedule of NHS Costs 2020/21 (Tab: Total Outpatient Attendance)^28^ |
| Day care service | Day care visit | £39 per client attendance | Local authority own-provision social services day care for adults requiring mental health support per client attendance | PSSRU 2021 (p.36)^25^ |

Note: all unit costs reported in the price year 2020-21. Abbreviations: A&E, accident and emergency; GP, general practitioner; NHS, National Health Service; PSSRU, Personal Social Services Research Unit.

Table S2. Health economics sensitivity analysis plan.

| Sensitivity analyses | Base case description | Sensitivity description | Source |
| --- | --- | --- | --- |
| A: Cost of Intervention, best case | £321 per person | £223 per person | Paterson et al (2022)^30^ |
| B: Cost of Intervention, worst case |  | £534 per person | Paterson et al (2022)^30^ |
| C: Cost of Intervention, no fixed cost |  | £251 per person | Paterson et al (2022)^30^ |
| D: Cost of Intervention, zero cost |  | £0 per person | Author assumption |
| E: QALY GLM, Poisson family | Gamma family | Poisson Family | Author assumption |
| F: Cost of non-mental health inpatient stay, per day payment | £368 per day | £291 per day | Average per day long stay payment for days exceeding the trim point 2020/21^31^ |
| G: Cost of non-mental health inpatient stay, zero cost | £368 per day | £0 per day | Author assumption |
| H: Multiple imputation, omit baseline variables | Baseline covariates: Age, Female Sex, MANSA, EQ-5D, Cost | Baseline covariates: EQ-5D, Cost | Author assumption |
| I: Complete case analysis | Multiple Imputation by Chained Equations | Available dataset | Author assumption |

Note: all unit costs reported in the price year 2020-21. Abbreviations: GLM, Generalised Linear Model; MANSA, Manchester Short Assessment of Quality of Life; QALY, Quality-adjusted life year.

# Appendix 7. Supplementary findings from the NEON Trial.

Table S3. Predictors of missingness for primary endpoint and interim outcomes

| Outcome | Baseline highest educational qualification p-value | Baseline MANSA p-value | Baseline hope p-value | Baseline mental health confidence scale p-value |
| --- | --- | --- | --- | --- |
| MANSA [week 52] | 0.0005 |  | 0.008 | 0.038 |
| MANSA [week 12] | 0.0005 | 0.032 |  |  |
| CORE-10 | 0.0005 |  | 0.028 |  |
| Herth Hope Index | 0.001 |  | 0.024 |  |
| Mental Health Confidence Scale | 0.0005 |  | 0.026 |  |
| Meaning in Life | 0.0005 |  |  | 0.026 |

Only significant p-values have been included. Two outcomes [*presence* and *search*] are calculated from subscales of the Meaning in Life Questionnaire data, but missingness is identical, and hence predictors of missingness are reported in a single row in this table. There were no significant predictors of missingness for interim MANSA data collected at week 1.

Table S4. Complete case clinical outcomes data.

|  | N | Control | Intervention | Baseline-adjusted difference  (95% CI) | p-value |
| --- | --- | --- | --- | --- | --- |
| MANSA [week 52] | 565 | 4·07 (1·0) | 4·13 (1·0) | 0·07 (-0·07 to 0·21) | 0·35 |
| **MANSA [week 12]** | 580 | 3·95 (1·0) | 4·01 (1·0) | 0·06 (-0·07 to 0·19) | 0·36 |
| **MANSA [week 1]** | 581 | 3·81 (1·0) | 3·85 (0·9) | 0·07 (-0·03 to 0·18) | 0·18 |
| **CORE-10** | 535 | 19·53 (7·9) | 19·83 (7·7) | -0·11 (-1·22 to 1·01) | 0·85 |
| **Herth Hope Index** | 534 | 31·28 (7·3) | 30·70 (6·7) | -0·41 (-1·39 to 0·57) | 0·41 |
| **Mental Health Confidence Scale** | 531 | 56·50 (16·5) | 56·27 (15·2) | 0·45 (-1·78 to 2·68) | 0·69 |
| **Meaning in Life: Presence subscale** | 531 | 3·89 (1·5) | 3·75 (1·4) | -0·11 (-0·31 to 0·09) | 0·28 |
| **Meaning in Life: Search subscale** | 531 | 4·51 (1·3) | 4·53 (1·4) | 0·03 (-0·16 to 0·23) | 0·74 |
| **EQ-5D-3L** | 523 | 0·55 (0·4-0·8) | 0·57 (0·4-0·8) | NA | NA |

Summary values are presented as mean (SD) for all measures other than for EQ-5D-3L, where we present median (IQR). No baseline-adjusted difference was calculated for EQ-5D-3L. Ranges for outcomes are MANSA [1 to 7]; CORE-10 [0 to 40]; Herth Hope Index [12 to 48]; Mental Health Confidence Scale [16 to 96]; Meaning in Life (presence and search subscales) [1 to 7]; EQ-5D-3L [‑0·59 to 1].

Table S5. Per-protocol analysis for the primary outcome at 52 weeks.

|  | Participants associated with protocol violation (n) | Participants in analysis (n) | Control  Mean (SD) | Intervention  Mean (SD) | Baseline adjusted difference (95% CI) | p-value |
| --- | --- | --- | --- | --- | --- | --- |
| Repeat registration for access to the intervention | 3 | 562 | 4·07 (1·0) | 4·14 (1·0) | 0·07 (-0·07 to 0·21) | 0·34 |
| Randomised in error | 1 | 564 | 4·07 (1·0) | 4·13 (1·0) | 0·07 (-0·07 to 0·21) | 0·34 |
| Allocated to control arm but had a period of intervention access before the primary endpoint | 1* | 565 | 4·07 (1·0) | 4·13 (1·0) | 0·07 (-0·07 to 0·21) | 0·35 |
| Primary outcome data provided late | 2 | 563 | 4·07 (1·0) | 4·14 (1·0) | 0·07 (-0·07 to 0·21) | 0·31 |
| At least one protocol violation p[resent | 7 | 559 | 4·07 (1·0) | 4·15 (1·0) | 0·08 (-0·07 to -0·22) | 0·29 |

*This participant did not have MANSA outcomes at 52 weeks and therefore had no impact on the analysis, results are identical to complete case in this situation

Table S6. Complete case analysis of 52-week clinical outcome data, adjusted for significant predictors of missingness.

|  | N | Baseline-adjusted difference (95% CI) | p-value |
| --- | --- | --- | --- |
| MANSA | 565 | 0·07 (-0·07 to 0·21) | 0·35 |
| CORE-10 | 535 | -0·03 (-1·13 to 1·07) | 0·96 |
| Herth Hope Index | 534 | -0·49 (-1·47 to 0·49) | 0·33 |
| Mental Health Confidence Scale | 531 | 0·32 (-1·90 to 2·53) | 0·78 |
| Meaning in Life: Presence subscale | 531 | -0·12 (-0·32 to 0·08) | 0·25 |
| Meaning in Life: Search subscale | 531 | 0·04 (-0·10 to -0·23) | 0·72 |

Table S7. p-values produced by an analysis of interaction effects with clinical outcomes at 52 weeks

|  | MANSA | CORE-10 | Hearth Hope Index | Mental Health Confidence Scale | Meaning in Life:  Presence subscale | Meaning in Life:  Search subscale |
| --- | --- | --- | --- | --- | --- | --- |
| Gender | 0·67 | 0·10 | 0·11 | 0·083 | 0·13 | 0·10 |
| Ethnicity | 0·94 | 0·84 | 0·74 | 0·36 | 0·16 | 0·80 |
| Have ever used specialist care mental health services | 0·10 | 0·45 | 0·31 | 0·10 | 0·51 | 0·25 |
| Current user of specialist care mental health services | 0·54 | 0·94 | 0·98 | 0·44 | 0·24 | 0·56 |

Analysis of the ITT sample (control n=369; intervention n=370) with missing data imputed.

Table S8. Exploratory analysis of 52-week outcome data, comparing intervention arm participants rating at least one narrative as “much more hopeful” with control arm participants.

|  | Control (N=639] | Intervention (N=87) | Baseline-adjusted difference (95% CI) | p-value |
| --- | --- | --- | --- | --- |
| MANSA | 4·07 (1·0) | 4·52 (0·9) | 0·34 (0·12 to 0·56) | 0·0026 |
| CORE-10 | 19·53 (7·9) | 16·33 (7·6) | -2·69 (-4·45 to -0·92) | 0·0029 |

Table S9. Analysis of the impact of national lockdown in England on baseline clinical outcomes.

|  | In national lockdown period N=289 | Not in national lockdown period N=450 | Difference  (95% CI) | p-value |
| --- | --- | --- | --- | --- |
| MANSA Mean (SD) | 3·75 (1·0) | 3·65 (0·9) | -0·10 (-0·24 to -0·03) | 0·138 |
| CORE-10 Mean (SD) | 22·80 (7·6) | 22·70 (7·2) | -0·10 (-1·10 to 1·10) | 0·984 |
| Herth Hope Index Mean (SD) | 29·00 (6·9) | 28·40 (6·7) | -0·60 (-1·70 to 0·40) | 0·210 |
| Mental Health Confidence Scale Mean (SD) | 49·70 (14·0) | 50·10 (14·4) | 0·40 (-1·70 to 2·60) | 0·675 |
| Meaning in Life: Presence subscale Mean (SD) | 3·55 (1·4) | 3·40 (1·4) | -0·15 (-0·36 to 0·06) | 0·167 |
| Meaning in Life: Search subscale Mean (SD) | 4·70 (1·3) | 4·56 (1·5) | -0·14 (-0·34 to 0·06) | 0·138 |

For each participant, the date of collection for a complete set of baseline MANSA items was used to code whether baseline data was collected in a national lockdown period or not. Difference was calculated as (not in lockdown-lockdown).

Table S10. Missing health economics data.

|  | **Total (N=739)** | **Control  (N=369)** | **Intervention (N=370)** |
| --- | --- | --- | --- |
| Health Outcomes, n (%) |  |  |  |
| EQ-5D-5L mobility, week 0 | 16 (2·2) | 10 (2·7) | 6 (1·6) |
| EQ-5D-5L self-care, week 0 | 16 (2·2) | 10 (2·7) | 6 (1·6) |
| EQ-5D-5L usual activities, week 0 | 16 (2·2) | 10 (2·7) | 6 (1·6) |
| EQ-5D-5L pain/discomfort, week 0 | 16 (2·2) | 10 (2·7) | 6 (1·6) |
| EQ-5D-5L anxiety/depression, week 0 | 16 (2·2) | 10 (2·7) | 6 (1·6) |
| EQ-5D-5L mobility, week 52 | 193 (26·1) | 84 (22·8) | 109 (29·5) |
| EQ-5D-5L self-care, week 52 | 193 (26·1) | 84 (22·8) | 109 (29·5) |
| EQ-5D-5L usual activities, week 52 | 193 (26·1) | 84 (22·8) | 109 (29·5) |
| EQ-5D-5L pain/discomfort, week 52 | 193 (26·1) | 84 (22·8) | 109 (29·5) |
| EQ-5D-5L anxiety/depression, week 52 | 193 (26·1) | 84 (22·8) | 109 (29·5) |
| *EQ-5D-3L, week 0* | *30 (4·1)* | *14 (3·8)* | *16 (4·3)* |
| *EQ-5D-3L, week 52* | *209 (28·3)* | *93 (25·2)* | *116 (31·4)* |
| *Total QALYs* | *216 (29·2)* | *96 (26·0)* | *120 (32·4)* |
| Resource use and Cost, n (%) |  |  |  |
| Mental health admissions, week 52 | 194 (26·3) | 85 (23·0) | 109 (29·5) |
| Non-mental health admissions, week 52 | 194 (26·3) | 85 (23·0) | 109 (29·5) |
| A&E visits, week 52 | 194 (26·3) | 85 (23·0) | 109 (29·5) |
| GP visits, week 52 | 194 (26·3) | 85 (23·0) | 109 (29·5) |
| Nurse visits, week 52 | 194 (26·3) | 85 (23·0) | 109 (29·5) |
| Psychologist visits, week 52 | 194 (26·3) | 85 (23·0) | 109 (29·5) |
| Psychiatrist visits, week 52 | 194 (26·3) | 85 (23·0) | 109 (29·5) |
| Day care visits, week 52 | 194 (26·3) | 85 (23·0) | 109 (29·5) |
| *Total cost, week 52* | *194 (26·3)* | *85 (23·0)* | *109 (29·5)* |

Italics indicate derived variables. Abbreviations: EQ-5D-5L, EuroQol 5-dimensions 5-levels; MANSA, Manchester Short Assessment of Quality of Life; QALY, Quality-adjusted life year.

Table S11. Cost-effectiveness sensitivity analyses.

| Analysis | Cost | | |  |  | QALYs | | | ICER |
| --- | --- | --- | --- | --- | --- | --- | --- | --- | --- |
|  | Intervention | Control | Incremental |  |  | Intervention | Control | Incremental |  |
| Adjusted base case | £3,465 | £2,288 | £1,177  (£438 to £1,969) |  |  | 0.5261 | 0.5154 | 0.0107  (-0.0041 to 0.0258) | £110,501 |
| A: Cost of Intervention, best case | £3,341 | £2,266 | £1,075  (£324 to £1,864) |  |  | 0.5260 | 0.5160 | 0.0100  (-0.0049 to 0.0252) | £107,339 |
| B: Cost of Intervention, worst case | £3,695 | £2,265 | £1,430  (£681 to £2,232) |  |  | 0.5261 | 0.5155 | 0.0106  (-0.0044 to 0.0255) | £134,982 |
| C: Cost of Intervention, no fixed cost | £3,315 | £2,277 | £1,038  (£295 to £1,830) |  |  | 0.5264 | 0.5154 | 0.0110  (-0.0038 to 0.0263) | £94,383 |
| D: Cost of Intervention, zero cost | £3,038 | £2,319 | £720  (-£30 to £1,494) |  |  | 0.5253 | 0.5144 | 0.0110  (-0.0043 to 0.0260) | £65,658 |
| E: QALY GLM, Poisson family | £3,528 | £2,323 | £1,205 (£442 to £2,009) |  |  | 0.5220 | 0.5166 | 0.0053 (-0.0101 to 0.0202) | £226,379 |
| F: Cost of non-mental health inpatient stay, per day payment | £3,411 | £2,229 | £1,181  (£461 to £1,926) |  |  | 0.5235 | 0.5147 | 0.0088  (-0.0064 to 0.0236) | £134,546 |
| G: Cost of non-mental health inpatient stay, zero cost | £3,192 | £2,117 | £1,075  (£440 to £1,697) |  |  | 0.5224 | 0.5145 | 0.0079  (-0.0071 to 0.0229) | £135,865 |
| H: Multiple imputation, omit baseline variables | £3,332 | £2,370 | £961  (£256 to £1,730) |  |  | 0.5292 | 0.5131 | 0.0161 (0.0007 to 0.0315) | £59,795 |
| I: Complete case analysis | £3,271 | £2,203 | £1,068 (£159 to £2,093) |  |  | 0.5410 | 0.5303 | 0.0107  (-0.0110 to 0.0319) | £99,565 |

Incremental results compare Intervention to Control (95% Bayesian credible interval for incremental outcomes reported in parentheses). ICER: Incremental cost-effectiveness ratio. QALY: Quality-adjusted life year.

Table S12. Cost-effectiveness analysis for service use subgroups.

| Analysis | Cost | | |  | QALYs | | | ICER |
| --- | --- | --- | --- | --- | --- | --- | --- | --- |
|  | Intervention | Control | Incremental |  | Intervention | Control | Incremental |  |
| 1: Have ever used specialist mental health services | £3,432 (n=309) | £2,437 (n=300) | £994 (£295 to £1,672) |  | 0.5222 (n=309) | 0.5111 (n=300) | 0.0111  (-0.0053 to 0.0276) | £89,352 |
| 2: Current user of specialist care mental health services | £3,888 (n=142) | £3,074 (n=137) | £814 (-£473 to £2,029) |  | 0.5383 (n=142) | 0.5150 (n=137) | 0.0232 (-0.0011 to 0.0471) | £35,013 |

Incremental results compare Intervention to Control (95% Bayesian credible interval for incremental outcomes reported in parentheses). ICER: Incremental cost-effectiveness ratio. QALY: Quality-adjusted life year.

Table S13. Distribution of responses to the mandatory narrative feedback question on hope promotion.

| **Less hopeful than before** | **No change** | **A bit more hopeful** | **Much more hopeful** |
| --- | --- | --- | --- |
| 178 (8·8%) | 767 (37·8%) | 770 (38·0%) | 313 (15·4%) |

Table S14. Number of participants accessing narratives outside of the NEON Intervention.

|  | Control  (N=369) | Intervention  (N=370) | Total  (N=739) |
| --- | --- | --- | --- |
| Week 1, n (%) |  |  |  |
| 0 | 168 (45·5) | 87 (23·5) | 255 (34·5) |
| 1-10 | 160 (43·4) | 224 (60·5) | 384 (52·0) |
| 11 or more | 6 (1·6) | 15 (4·1) | 21 (2·8) |
| Missing | 35 (9·5) | 44 (11·9) | 79 (10·7) |
| Week 12, n (%) |  |  |  |
| 0 | 121 (32·8) | 55 (14·9) | 176 (23·8) |
| 1-10 | 173 (46·9) | 199 (53·8) | 372 (50·3) |
| 11 or more | 14 (3·8) | 48 (13·0) | 62 (8·4) |
| Missing | 61 (16·5) | 68 (18·4) | 129 (17·5) |
| Week 52, n (%) |  |  |  |
| 0 | 126 (34·1) | 67 (18·1) | 193 (26·1) |
| 1-10 | 132 (35·8) | 142 (38·4) | 274 (37·1) |
| 11 or more | 26 (7·0) | 52 (14·1) | 78 (10·6) |
| Missing | 85 (23·0) | 109 (29·5) | 194 (26·3) |


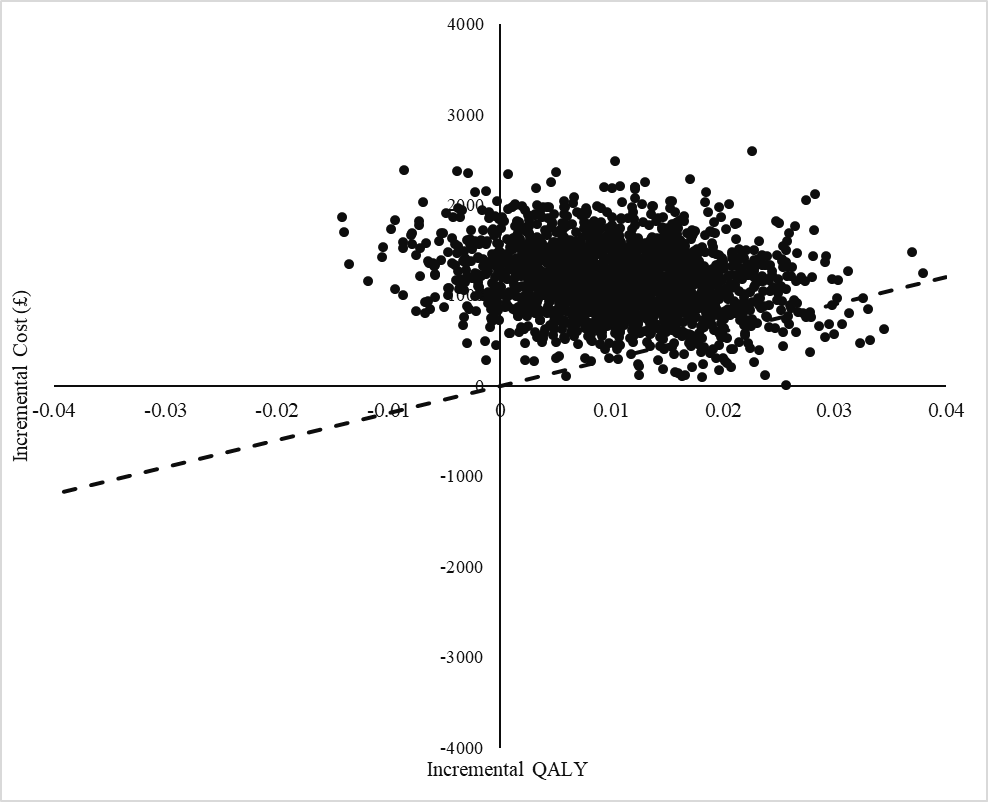


Figure S4. Cost-effectiveness plane calculated for the adjusted base case analysis.

Analysis of the ITT sample (control n=369; intervention n=370) with missing data imputed. Dashed line represents a cost-effectiveness threshold of £30,000 per quality adjusted life year (QALY) gained.


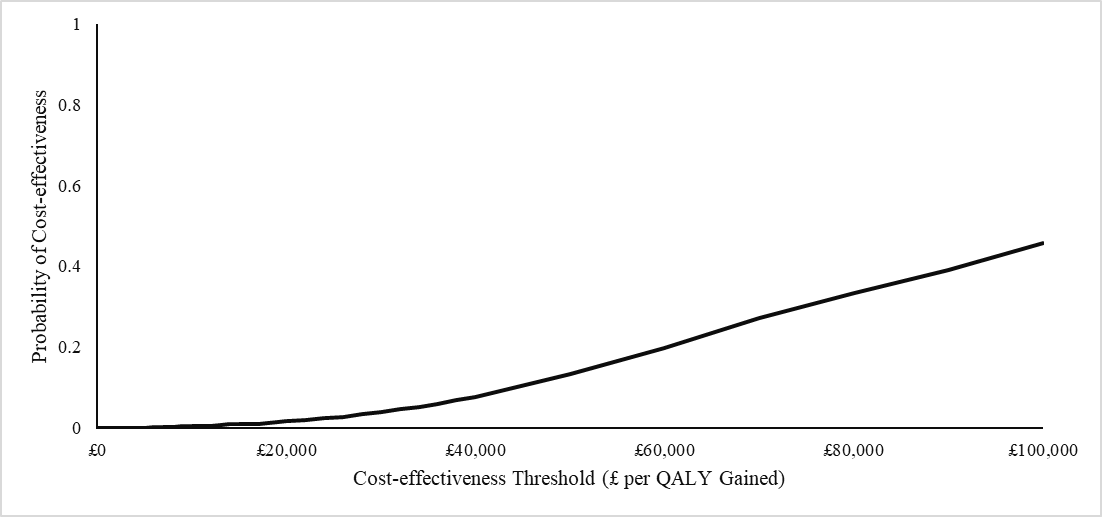


Figure S5. Cost-effectiveness acceptability curve for the adjusted base case analysis.

Analysis of the ITT sample (control n=369; intervention n=370) with missing data imputed.

# References for all appendices.

1. Schulz KF, Altman DG, Moher D. CONSORT 2010 statement: updated guidelines for reporting parallel group randomised trials. Trials. 2010;11(1):1-8.

2. Husereau D, Drummond M, Augustovski F, de Bekker-Grob E, Briggs AH, Carswell C, et al. Consolidated Health Economic Evaluation Reporting Standards 2022 (CHEERS 2022) statement: updated reporting guidance for health economic evaluations. BMJ. 2022;376:e067975.

3. Smith R. Stop Paddling/Start Sailing. Maryland: America Star Books; 2004.

4. Yeo C, Hare-Duke L, Rennick-Egglestone S, Bradstreet S, Callard F, Hui A, et al. The VOICES typology of curatorial decisions in narrative collections of the lived experiences of mental health service use, recovery, or madness: qualitative study. JMIR mental health. 2020;7(9):e16290.

5. Yeo C, Rennick Egglestone S, Armstrong V, Borg M, Charles A, Hare Duke L, et al. The influence of curator goals on collections of lived experience narratives: Qualitative study. Journal of Recovery in Mental Health. 2021;Summer 2021.

6. Slade M, Rennick-Egglestone S, Llewellyn-Beardsley J, Yeo C, Roe J, Bailey S, et al. Recorded mental health recovery narratives as a resource for people affected by mental health problems: development of the Narrative Experiences Online (NEON) Intervention. JMIR Formative Research. 2021;5(5):e24417.

7. Kotera Y, Rennick-Egglestone S, Ng F, Llewellyn-Beardsley J, Ali Y, Newby C, et al. Assessing diversity and inclusivity is the next frontier in mental health recovery narrative research and practice. JMIR Mental Health. 2023;10(1):e44601.

8. Linton S, R W, editors. The Colour of Madness. Basingstoke, UK: Pan Macmillan; 2022.

9. Riding the Storms. Devon: Recovery Devon; 2019.

10. No surname S. What helped or made things worse [Available from: <https://www.youtube.com/embed/XmH1632Gm9A>.

11. Shallowhorn K. From Mental Health Institutionalization to Advocacy [Available from: <https://oc87recoverydiaries.org/mental-health-institutionalization/>.

12. Cordle H, Carson J, Richards P, Fradgley J. Psychosis: Stories of Recovery and Hope. Salisbury: Quay Books; 2010.

13. Llewellyn-Beardsley J, Barbic S, Rennick-Egglestone S, Ng F, Roe J, Hui A, et al. INCRESE: Development of an Inventory to Characterize Recorded Mental Health Recovery Narratives. Journal of Recovery in Mental Health. 2020;3(2).

14. Charles A, Hare-Duke L, Nudds H, Franklin D, Llewellyn-Beardsley J, Rennick-Egglestone S, et al. Typology of content warnings and trigger warnings: Systematic review. PloS one. 2022;17(5):e0266722.

15. Llewellyn-Beardsley J, Rennick-Egglestone S, Callard F, Crawford P, Farkas M, Hui A, et al. Characteristics of mental health recovery narratives: systematic review and narrative synthesis. PloS One. 2019;14(3):e0214678.

16. Llewellyn-Beardsley J, Rennick-Egglestone S, Bradstreet S, Davidson L, Franklin D, Hui A, et al. Not the story you want? Assessing the fit of a conceptual framework characterising mental health recovery narratives. Social psychiatry and psychiatric epidemiology. 2020;55(3):295-308.

17. Llewellyn-Beardsley J, Rennick-Egglestone S, Pollock K, Ali Y, Watson E, Franklin D, et al. ‘Maybe I Shouldn’t Talk’: The Role of Power in the Telling of Mental Health Recovery Stories. Qualitative Health Research. 2022;32(12):1828-42.

18. Rennick-Egglestone S, Morgan K, Llewellyn-Beardsley J, Ramsay A, McGranahan R, Gillard S, et al. Mental Health Recovery Narratives and Their Impact on Recipients: Systematic Review and Narrative Synthesis. Canadian Journal of Psychiatry. 2019;64(10).

19. Rennick-Egglestone S, Ramsay A, McGranahan R, Llewellyn-Beardsley J, Hui A, Pollock K, et al. The impact of mental health recovery narratives on recipients experiencing mental health problems: qualitative analysis and change model. PloS one. 2019;14(12):e0226201.

20. Ng F, Newby C, Robinson C, Llewellyn-Beardsley J, Yeo C, Roe J, et al. How do recorded mental health recovery narratives create connection and improve hopefulness? Journal of Mental Health. 2022;31(2):273-80.

21. Rennick-Egglestone S, Elliott R, Smuk M, Robinson C, Bailey S, Smith R, et al. Impact of receiving recorded mental health recovery narratives on quality of life in people experiencing psychosis, people experiencing other mental health problems and for informal carers: Narrative Experiences Online (NEON) study protocol for three randomised controlled trials. Trials. 2020;21(1):1-34.

22. Rennick-Egglestone S, Knowles S, Toms G, Bee P, Lovell K, Bower P. Health Technologies' In the Wild': Experiences of Engagement with Computerised CBT. Proceedings of the 2016 CHI Conference on Human Factors in Computing Systems. 2016:2124-35.

23. Slade M, Rennick-Egglestone S, Llewellyn-Beardsley J, Yeo C, Roe J, Bailey S, et al. Recorded mental health recovery narratives as a resource for people affected by mental health problems: development of the Narrative Experiences Online (NEON) intervention. JMIR formative research. 2021;5(5):e24417.

24. Slade E, Rennick-Egglestone S, Ng F, Kotera Y, Llewellyn-Beardsley J, Newby C, et al. The Implementation of Recommender Systems for Mental Health Recovery Narratives: Evaluation of Use and Performance. JMIR Mental Health.11.

25. Jones KC, Burns A. *Unit Costs of Health and Social Care 2021*. Canterbury: Personal Social Services Research Unit; 2021. Report No.: 9781911353140.

26. NHS Improvement. *Reference costs 2017/18: Highlights, Analysis and Introduction to the Data*. London; 2018.

27. Jones KC, Weatherly H, Birch S, Castelli A, Chalkley M, Dargan A, et al. *Unit Costs of Health and Social Care 2022 Manual*. Canterbury: Personal Social Services Research Unit (University of Kent) & Centre for Health Economics (University of York); 2023.

28. NHS England. National Schedule of NHS Costs 2020/21 [Available from: <https://www.england.nhs.uk/publication/2020-21-national-cost-collection-data-publication/>.

29. Curtis L, Burns A. *Unit Costs of Health and Social Care 2015*. Canterbury: Personal Social Services Research Unit; 2015. Report No.: 978-1-902671-96-3.

30. Paterson L, Rennick-Egglestone S, Gavan SP, Slade M, Ng F, Llewellyn-Beardsley J, et al. Development and delivery cost of digital health technologies for mental health: Application to the Narrative Experiences Online Intervention. Frontiers in Psychiatry. 2022;13.

31. NHS England. National Tariff 2020/21: Documents and Policies. 2021 [Available from: <https://www.england.nhs.uk/publication/past-national-tariffs-documents-and-policies/>.
